# Smart and Functionalized Development of Nucleic Acid‐Based Hydrogels: Assembly Strategies, Recent Advances, and Challenges

**DOI:** 10.1002/advs.202100216

**Published:** 2021-05-07

**Authors:** Yangzi Zhang, Longjiao Zhu, Jingjing Tian, Liye Zhu, Xuan Ma, Xiaoyun He, Kunlun Huang, Fazheng Ren, Wentao Xu

**Affiliations:** ^1^ Key Laboratory of Precision Nutrition and Food Quality Department of Nutrition and Health China Agricultural University No. 17, Qinghua East Road Beijing 100083 China; ^2^ Key Laboratory of Safety Assessment of Genetically Modified Organism (Food Safety) (MOA) College of Food Science and Nutritional Engineering China Agricultural University No. 17, Qinghua East Road Beijing 100083 China; ^3^ Beijing Laboratory for Food Quality and Safety College of Food Science and Nutritional Engineering China Agricultural University No. 17, Qinghua East Road Beijing 100083 China

**Keywords:** biosensing, drug delivery, functional nucleic acid, nucleic acid‐based hydrogel, stimuli‐responsive hydrogel

## Abstract

Nucleic acid‐based hydrogels that integrate intrinsic biological properties of nucleic acids and mechanical behavior of their advanced assemblies are appealing bioanalysis and biomedical studies for the development of new‐generation smart biomaterials. It is inseparable from development and incorporation of novel structural and functional units. This review highlights different functional units of nucleic acids, polymers, and novel nanomaterials in the order of structures, properties, and functions, and their assembly strategies for the fabrication of nucleic acid‐based hydrogels. Also, recent advances in the design of multifunctional and stimuli‐responsive nucleic acid‐based hydrogels in bioanalysis and biomedical science are discussed, focusing on the applications of customized hydrogels for emerging directions, including 3D cell cultivation and 3D bioprinting. Finally, the key challenge and future perspectives are outlined.

## Graphical abstract

Nucleic acid‐based hydrogels present the trend of interdisciplinary development from the diversified construction building blocks to the extensive application fields, mainly including biosensing, drug delivery, 3D cell cultivation, 3D bioprinting, bioimaging, and treatment and analysis of environmental pollutants.

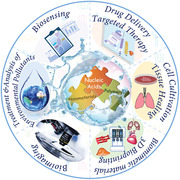

## 1 Introduction

**Figure 1:**
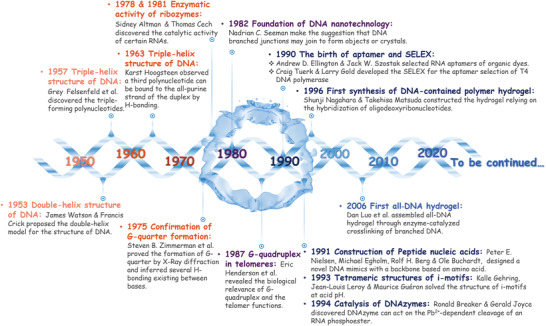
Memorabilia of FNAs promoting the development of DNA nanotechnology and the birth of NAHs.

Over nearly four decades of development, DNA nanotechnology has driven the generation and progress of a cluster of subdisciplines, especially taking a big step in the biomaterial science direction^[^ ^1^ ^]^ (**Figure** **1**). In the early 1980s, the groundbreaking study of Seeman established a solid foundation for DNA nanotechnology by constructing controllable new nanoscale structures and mechanisms based on the molecule properties of DNA.^[^ ^2^, ^3^ ^]^ Since then, DNA has not only been regarded as the blueprint of life but also as a universal element for creating nanoscale structures and materials. The subfields from the initial structural DNA nanotechnology to the recent functional DNA nanotechnology are entirely different. However, they infiltrated mutually and developed crosswise at the same time, leading to the formation of the pattern of unity in diversity. Accordingly, a series of novel materials have emerged, based on DNA assembly, such as DNA origami, nanoparticle–DNA colloidal materials, and polymer–DNA materials.^[^ ^1^, ^4^ ^]^ These products possess unique DNA nanostructures and molecular properties and are integrated with other polymers and nanomaterials to meet the developmental demand for DNA nanotechnology and applications in material engineering.

The advancement of DNA synthesis techniques, the innovation of precise DNA nanostructures, and the insight into DNA characteristics have actively increased the necessity for novel nucleic acid‐based biomaterials.^[^ ^5^, ^6^, ^7^, ^8^, ^9^, ^10^ ^]^ From the birth of the first DNA‐containing polymer hydrogels in 1996^[^ ^11^ ^]^ to the successful development of self‐supporting pure DNA hydrogel in 2006,^[^ ^12^ ^]^ the versatility of DNA has inspired an interest in hydrogel design, such as strong hydrophilicity, biocompatibility, structural programmability, controlled biodegradability, nutrient permeability, adjustable mechanical properties, stability against proteases, self‐healing ability, and stimuli‐responsiveness to the hydrogel.^[^ ^13^, ^14^, ^15^, ^16^ ^]^ However, the investigation of DNA‐based hydrogels was at a preliminary stage with limited studies at that time, of which only a small part involved the stimuli‐triggered responses.^[^ ^17^ ^]^ Since 2004, the development and improvement of functional features have become a new research focus of DNA‐based hydrogels, especially the vigorous development of exceptionally high‐profile stimuli responsiveness by implementing different FNAs. Furthermore, partial studies have been devoted to exploring the mechanical properties of DNA‐based hydrogels and their effects. With an increased curiosity and demand for these novel soft biomaterials, the application orientation for smart nucleic acid‐based hydrogels (NAHs) is gradually becoming clear, such as utilizing them for tissue engineering, drug, delivery, and biosensing.^[^ ^18^ ^]^

We frequently focus on the development and application of FNAs and related nanomaterials. FNAs are a class of natural or artificial nucleic acid sequences with independent structures and specific biological functions.^[^ ^19^ ^]^ In 2019, we successively summarized the development, properties, and applications of FNA–nanomaterials,^[^ ^20^ ^]^ as well as the principles and methods of FNA tailoring strategies.^[^ ^21^ ^]^ For future studies, we explicated two main challenges. First, the creation of accurate and efficient FNA–nanomaterials can be a powerful tool in understanding life processes, detecting various biomarkers, and treating diseases at a high level, which must be based on the comprehensive study of the essence of the interaction between FNAs and nanomaterials, as well as their application abilities.^[^ ^20^ ^]^ Second, the construction of sophisticated structures, novel 3D DNA patterns, and concise biosensors relies on the progress of FNA tailoring strategies in optimizing the functions of FNA sequences, targeting and tracking drug release in tumors, imaging‐guided focal therapy, and the monitoring of therapeutic responses.^[^ ^21^ ^]^ Furthermore, a series of studies of smart biomaterials including NAHs are emerging rapidly to keep abreast of the development of DNA nanotechnology and nanomaterials. By definition of Newnham and Ruschau,^[^ ^22^ ^]^ smart materials refer to a class of materials that can perform both sensing and actuating functionality. It is different with intelligent materials that smart materials are not equipped with the control system and the information processing function.^[^ ^22^, ^23^ ^]^ Thus, developed smart NAHs at a current stage which provides automatic feedback based on their inherent structure, properties, and perception of the environment not dependently on computer big data for synthesis, belong to smart materials. Nowadays, the continuously‐updated structures and functional units of nucleic acids and other polymers or materials contribute significantly to the success of developing smart NAHs. In the meanwhile, researchers pay more attention to how to coordinate various functions in the NAHs, while exploring diverse stimuli for triggering responses in practical applications. It is believed that the answer will be found in developing smart NAHs with functionalized integration and sensitive responsiveness. Therefore, it is necessary to review recent research and development involving NAHs and to propose appropriate strategies for a combination of functionalized DNA nanomaterials with stimuli‐responsive NAHs.

This review summarizes recent advances in the smart and functionalized development of NAHs, following the order of structures, properties, and functions. Different functional units, including FNAs, polymers, and nanomaterials, are introduced, and their assembly strategies regarding the use of nucleic acids in the hydrogels are discussed. In contrast to other recent reviews on DNA hydrogels, this work explicitly summarizes the functional features and stimuli‐responsive strategies of NAHs to provide new dimensions and promising perspectives for their design and development. Specifically, many applications are introduced involving NAHs that are customized with desirable functions and smart features in emerging fields, such as tissue engineering, drug delivery, controlled release, and biosensing. Considering the market demand and the current limitations, the DNA‐based smart hydrogels are evaluated for prospective future use.

## 2 Functional Units and Their Assembly Strategies for NAHs

The diversity and application potentiality of NAHs are primarily reflected by the utilization, collocation, and assembly of functional units. With the rational and well‐designed assembly strategies, these units can maximize versatility and even break or alleviate mutual limitations but remain respective key features. The integrated smart components in various developed NAHs primarily include FNAs, polymers, and nanomaterials. These smart and functional components utilized in the construction strategies will effectively upgrade functionality and expand the applicability of NAHs from two perspectives: one is to incorporate different FNAs as the building blocks of backbones or as the cross‐linkers for the synthesis of hydrogels, which provide additional molecular recognition, catalytic activity, and therapeutic potential; the other one is to construct hybrid NAHs with other synthetic polymers or nanomaterials, such as graphene oxide (GO), metal nanoparticles, and magnetic beads, which combines the advantages of two or more materials to improve the functionality. In this section, the typical smart components are introduced, while the assembly strategies aimed at the intelligence improvement of NAH are addressed from three viewpoints based on physical and chemical cross‐linking methods, namely nucleic acid amplification techniques, click reactions, and macrocyclic guest–host interactions.

### 2.1 FNAs

**Figure 2:**
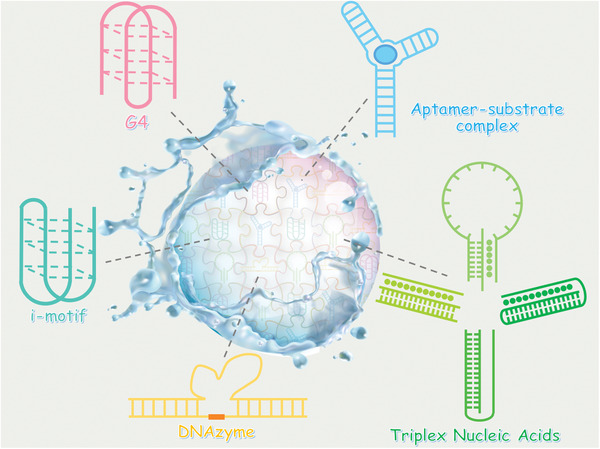
Schematic illustration of key FNAs units in DNA hydrogels.

FNAs are regarded as excellent substitutes for traditional proteases and antibodies due to their superior features, which include easy modification, low cost, high stability, and strong specificity. FNAs mainly include aptamers, catalytic nucleic acids (deoxyribozymes or ribozymes), triplex nucleic acids, peptide nucleic acids (PNA), G‐quadruplex (G4), i‐motifs, and metallo‐nucleic acid complexes, which exhibit molecular recognition, catalytic activity, and multiple functions under hydrogen bonds, Van Der Waals forces, and hydrophobic interactions.^[^ ^24^, ^25^, ^26^, ^27^ ^]^ According to the reported studies, aptamer, G4, i‐motifs, and triplex nucleic acids have become key players in developing smart and functionalized DNA hydrogels for bioanalysis and biomedical applications (**Figure** **2**).

Aptamers are single‐stranded oligonucleotides that are obtained by screening from libraries of random nucleic acid sequences in vitro using the SELEX technique.^[^ ^24^ ^]^ The binding capability of aptamers toward the specific targets is essentially decided by their functional secondary structures, which endow aptamers with high affinity, thereby providing the highly‐targeted ability for binding corresponding substrates.^[^ ^28^ ^]^ Even though substrates are at exceedingly low concentrations or with changes in structure, the high specificity of aptamers also effectively ensure the accurate identification of substrates and binding between the two.^[^ ^29^ ^]^ Thus, these aptamers have been widely used to stimulate the DNA‐based hydrogel system to induce specific physical or chemical changes. The interactions between aptamers and specific targets are determined by the equilibrium relationship between molecular association and dissociation, which is typically represented as dissociation constants (*K*_d_).^[^ ^28^ ^]^ *K*_d_ is a key index to evaluate the affinity between the aptamer and its corresponding target. Generally, *K*_d_ values of most aptamers are relatively low, ranging from nanomolar (nM) to picomolar (pM) level. In addition, the superiorities of aptamers also reflect in easy chemical modification, and highly specific binding again after dissociation with substrates. At the same time, the high stability of aptamers enables multiple cycles between thermal denaturation and renaturation without losing their binding ability. Thus, DNA‐based hydrogels incorporated with aptamers provide ideal biorecognition elements for biosensors.^[^ ^30^, ^31^, ^32^ ^]^ Furthermore, since the low molecular weight of aptamers promotes the probability of their excretion via blood, they are protected effectively from the degradation and delivered to the specific sites for the rapid and accurate identification of targets in vivo by being embedded on the scaffolds or being directly encapsulated into the hydrogels. Currently, aptamers are widely used for molecular detection and medical diagnosis.^[^ ^33^, ^34^ ^]^ A study by Li et al.^[^ ^19^, ^35^ ^]^ showed that a stimuli‐responsive hydrogel that self‐assembled with specific aptamers effectively inhibited the proliferation of target A549 tumor cells and controlled their migration, which restricted the potentiality of using aptamers in targeted gene therapy. Similarly, Zhou et al.^[^ ^36^ ^]^ used ochratoxin A (OTA) aptamers as DNA linkers to hybridize with scaffolds of hydrogel to form target‐dependent switchable DNA hydrogels, providing space for encapsulating the signal molecule horseradish peroxidase (HRP). The association between the OTA target and its specific aptamer encouraged the hydrogel to release HRP while facilitating visualization detection and sensitive colorimetric analysis.

Deoxyribozymes (DNAzymes), composed of a substrate chain and an enzyme chain, are typical FNAs obtained via in vitro screening.^[^ ^25^ ^]^ As a kind of biocatalyst, the most important attribute of DNAzymes is specific binding/cleavage ability and high catalytic activity. Generally, the enzyme chain can quickly cleave the specific site of the substrate chain with the assistance of metal ions or amino acids acting as co‐catalysts, while the cleavage efficiency is affected by the DNAzyme concentration and the number of sequences binding on the substrate surface.^[^ ^37^, ^38^ ^]^ Moreover, DNAzymes can catalyze a series of reactions, including phosphodiester bond hydrolysis and peroxide degradation. The catalytic efficiency can be improved via effective allosteric control by oligonucleotides or small molecules.^[^ ^37^ ^]^ Therefore, DNAzymes integrated into DNA hydrogels endow biomaterials with catalytic abilities and molecular recognition functions. Furthermore, to develop native biocatalytic mimic pathways using enzyme cascades, Xiang et al.^[^ ^39^ ^]^ integrated a peroxidase‐mimicking DNAzyme as a DNA motif within scaffolds to construct a functional DNAzyme hydrogel. Subsequent coencapsulation of glucose oxidase (GOx) and *β*‐galactosidase into this biomaterial allowed for the successful construction of a hybrid enzymatic cascade reaction system to achieve visual glucose/lactose detection.

G4 are four‐stranded structures comprising stacked G‐tetrads that are formed by the self‐assembly of G‐rich nucleic acid sequences containing the tandem nucleobase, guanine.^[^ ^40^ ^]^ In the presence of specific ions, such as K^+^, Pb^2+^ or NH_4_^+^, G4 can form parallel or antiparallel stable structures according to the orientation of the four strands. G4 not only possesses the high stability and biocompatibility of G4 under physiological conditions but also displays both the fluorescent property binding with small molecules and peroxidase‐mimicking activity resulting from hemin. Thus, it is appropriate to be incorporated as the functional DNA motif into DNA nanomaterials and has been widely applied in the biological colorimetric analysis.^[^ ^36^ ^]^ Huang et al.^[^ ^41^ ^]^ obtained a dense G4 interchain structure using rolling circle amplification (RCA) to prepare a functional DNA hydrogel system, which encapsulated GOx for glucose detection. The results showed that the hydrogel environment containing the G4 structure exhibited excellent sensitivity and stability.

The i‐motif is a special cytosine‐rich tetraplex structure. It is formed by two parallel duplexes assembling in antiparallel under weak acidic conditions, while each parallel duplex is composed of two cytosine chains binding through C·CH^+^ pairings.^[^ ^27^ ^]^ The stability of the i‐motif structure is affected by the length of the cytosine region, sequence length, temperature, salt concentration, and the most critical, environmental pH. The i‐motif generally remains stable in the optimum range of pH 3–7.^[^ ^42^, ^43^ ^]^ Based on the pH dependence of the i‐motif structure, it is ideal for constructing pH‐responsive DNA hydrogels, while achieving a rapid response or cross‐linking by adjusting the pH levels. The transformation of the i‐motif structure at different pH values enables the DNA hydrogel to rapidly change morphology, making it ideal for application in bioanalysis and biomedical fields.^[^ ^44^ ^]^

Similarly, triplex nucleic acids, such as the C‐G·C or T‐A·T triplexes, are another type of FNAs and their formation is also driven by changes in pH levels. An acidic pH environment promotes the protonation of cytosine, allowing the formation of a stable C‐G·C triplex structure, while a neutral pH environment promotes the formation of a T‐A·T triplex. Consequently, stable triplex nucleic acids supported by specific triplex structures can be generated in certain pH conditions.^[^ ^45^ ^]^ This explicit pH‐responsiveness has been incorporated into the design of smart responsive DNA hydrogels. In 2015, the team of Willner first presented the pH‐responsive and switchable triplex‐based DNA hydrogels. At appropriate pH values, two hydrogel systems based on the C‐G·C or T‐A·T triplexes achieved the reversible cycles of hydrogel/solution transition, which were applied for the loading and release of the anti‐cancer drug, coralyne.^[^ ^46^ ^]^ Using triplexes as bridging units in the acrylamide (AM)/DNA hydrogels in subsequent studies, facilitated the development of several functional hydrogel matrices, which were stabilized with either a combination of pH‐responsiveness and shape memory properties^[^ ^47^ ^]^ or with dual‐shape‐memory systems.^[^ ^48^ ^]^ Furthermore, the triplex structures have also been utilized to cross‐link Y‐shaped building blocks for the reversible self‐assembly of pure DNA hydrogel in specific pH conditions, improving the biocompatibility of nucleic acid‐based hydrogels applied for diagnosis, treatment, and medicine.^[^ ^49^ ^]^ Therefore, the nucleic‐acid‐based hydrogels embedded with triplex structures exhibit significant promise for utilization in biomedicine.

PNA is a special type of FNAs which is artificially synthesized with a neutral peptide‐like backbone by replacing the phosphodiester backbone with 2‐aminoethyl‐glycine linkage.^[^ ^50^ ^]^ Such a structure endows PNAs with superior hybridization behavior and high biological stability due to lack of charge repulsion.^[^ ^50^, ^51^ ^]^ Thus, PNAs are appropriate to participate in the assembly of nucleic acid‐based biomaterials and suitable for biomedical applications. In the meanwhile, a series of previous studies have indicated that PNAs specifically reorganize and bind with DNA to form different helix structures obeying the Watson‐Crick pattern or under the Watson‐Crick and Hoogsteen base interactions such as duplex, triplex, or even quadruplex conformation.^[^ ^52^, ^53^ ^]^ This evidence provides more possibilities for the construction of hybrid NAHs, which has been confirmed by Chu et al. They developed two types of double‐helix and triple‐helix stabilized hybrid NAHs by cross‐linking DNA linkers with the linear N‐(2‐hydroxypropyl) methacrylamide (HPMA) polymers grafted with multiple PNAs. According to a series of characterization on these two hybrid NAHs, the results demonstrated that the introduction of PNAs can form the interconnected microporous network and was beneficial to elevate the rigidity and cross‐linking density of hybrid NAHs with the increase of grafted PNAs. Furthermore, these PNA‐based hydrogels showed promise as delivery vehicles for drug or gene delivery, as valves for microfluidic devices, or directly as the tissue scaffolds for biomedical engineering.^[^ ^53^ ^]^

### 2.2 Polymers and Nanomaterials

Polymers and nanomaterials are essential components to drive the innovation and development of hybrid NAHs. This actively synergistic effect significantly improves the mechanical strength of biomaterials, accelerates the assembly of NAHs, reduces the required concentration of nucleic acids, and further broadens the application scopes of these biomaterials.^[^ ^54^, ^55^, ^56^ ^]^

Currently, the synthetic polymers used for constructing DNA‐polymer hybrid hydrogels mainly include polyacrylamide (PAM),^[^ ^57^, ^58^, ^59^, ^60^, ^61^ ^]^ poly‐*N*‐isopropylacrylamide (pNIPAM),^[^ ^62^, ^63^, ^64^ ^]^ polyethyleneimine (PEI),^[^ ^65^ ^]^ and polyethylene glycol (PEG).^[^ ^66^, ^67^, ^68^ ^]^ In addition, poly‐L‐lysine (PLL),^[^ ^69^, ^70^ ^]^ carboxymethyl cellulose (CMC),^[^ ^71^, ^72^ ^]^ and other high‐molecular polymers obtained via polymerization or by modifying natural raw materials can also work as the backbone or cross‐linkers in the hybrid DNA hydrogels. The participation of synthetic polymers in the hydrogel not only substantially reduces the use of nucleic acids while saving costs, but also increases the mechanical strength and stabilizes the hydrogel network via the electrostatic interaction with DNA.^[^ ^54^ ^]^ Moreover, the incorporated synthetic polymers do not affect the original properties of the DNA itself, endowing the hydrogel with multiple functionalities.^[^ ^54^ ^]^ For example, pNIPAM is a thermosensitive polymer that can result in the shrinking or swelling of the hydrogel, while further inducing the phase change by adjusting the lowest critical solution temperature (LCST)‐based temperature. Based on this reversible change in pNIPAM, Li et al.^[^ ^63^ ^]^ prepared a pNIPAM‐DNAzyme hydrogel that displayed intense catalytic activity in the liquid phase system at room temperature. While above the LCST, the hydrogel exhibited a hydrophobic effect after undergoing a phase change and separated from the reaction system to achieve DNAzyme recycling. However, compared with pure DNA hydrogels, these hybrid DNA‐based hydrogels may display lower biocompatibility which is mainly impacted using toxic reagents for the modification and coupling of DNA and functional materials such as polymers and nanoparticles.^[^ ^49^, ^73^ ^]^ The residual toxic reagents in the hydrogel system limit applicability of hybrid DNA‐based hydrogels in cell transplantation and encapsulation, the production of proteins, and drug delivery in vivo.^[^ ^55^, ^73^, ^74^, ^75^ ^]^

Another type of hybrid hydrogel consists of DNA–nanomaterial and is primarily assembled in one of two ways. Either the cross‐linking between the DNA building blocks and nanomaterials is formed via physical interaction, or the nanomaterials are encapsulated in the DNA hydrogel networks. The nanomaterials applied for the development of hybrid NAHs cover a variety of categories, including carbon‐based nanomaterials, such as GO,^[^ ^76^, ^77^ ^]^ carbon‐nanotubes,^[^ ^78^, ^79^ ^]^ carbon dots,^[^ ^80^, ^81^, ^82^ ^]^ quantum dots,^[^ ^83^ ^]^ magnetic beads,^[^ ^84^, ^85^, ^86^, ^87^, ^88^ ^]^ liposome,^[^ ^57^ ^]^ gold nanoparticles (AuNPs),^[^ ^68^, ^75^, ^89^, ^90^, ^91^ ^]^ silica nanoparticles,^[^ ^54^, ^92^ ^]^ Laponite nanoparticles,^[^ ^93^, ^94^, ^95^ ^]^ and photonic crystal (PC).^[^ ^96^ ^]^ The interaction between DNA and nanomaterials reveals their multiple functional features, further enhancing the corresponding applications. For example, a GO/DNA composite hydrogel was developed where the negatively charged DNA hydrogel displayed improved GO conductivity, which can be used as a highly sensitive electrochemical biosensor for bioanalysis.^[^ ^97^ ^]^ Similarly, Zhang et al.^[^ ^83^ ^]^ reported a self‐assembled quantum dot DNA hydrogel for enzyme‐responsive drug delivery and cell‐specific targeting. The multifunctionality of this hydrogel was achieved by leveraging DNA‐guided interactions and DNA‐templated quantum dots with high quantum yields. Furthermore, in the DNA/clay hydrogels, the clay nanoparticles acted as a chemical cross‐linker to promote their elasticity, whereas the DNA contributed to their viscoelastic energy dissipation. This cooperative relationship between the clay nanoparticles and DNA provided the hydrogels with high elasticity and self‐healing properties.^[^ ^94^ ^]^ Moreover, the cooperation between nucleic acids and nanomaterials in the hydrogel materials inspires a superposition effect in the functional features, promoting the use of hybrid NAHs in a variety of applications.

### 2.3 Assembly Strategies of the Functional Components

**Figure 3:**
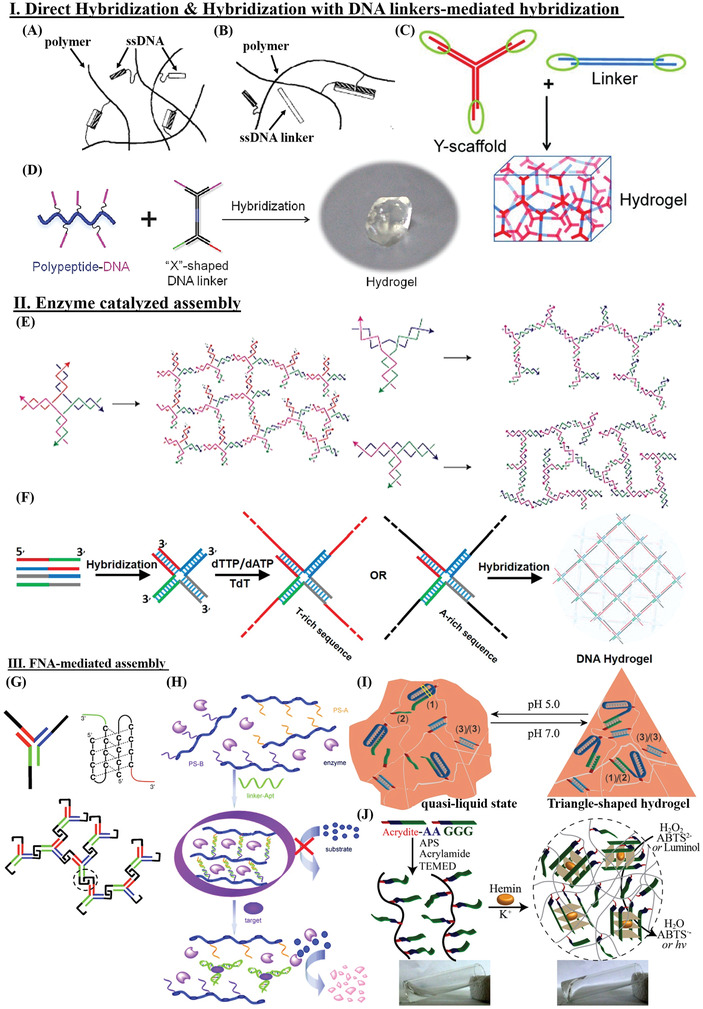
General scheme of DNA‐based hydrogels assembly by A) direct hybridization, B) ssDNA linker‐mediated hybridization,^[^ ^11^ ^]^ C) dsDNA linker‐mediated hybridization,^[^ ^100^ ^]^ D) “X”‐shaped DNA linker mediated hybridization,^[^ ^101^ ^]^ E) T4 DNA ligase‐meditated catalysis,^[^ ^12^ ^]^ F) TdT‐mediated catalysis,^[^ ^39^ ^]^ G) i‐motif as cross‐linker,^[^ ^102^ ^]^ H) aptamer as cross‐linker,^[^ ^90^ ^]^ I) C‐G·C^+^ triplex as cross‐linker,^[^ ^47^ ^]^ J) G4 as cross‐linker.^[^ ^103^ ^]^ A,B) Adapted with permission.^[^ ^11^ ^]^ Copyright 1996, Elsevier B.V. C) Adapted with permission.^[^ ^100^ ^]^ Copyright 2011, Wiley‐VCH. D) Adapted with permission.^[^ ^101^ ^]^ Copyright 2014, Wiley‐VCH. E) Adapted with permission.^[^ ^12^ ^]^ Copyright 2006, Springer Nature. F) Adapted with permission.^[^ ^39^ ^]^ Copyright 2016, American Chemical Society. G) Adapted with permission.^[^ ^102^ ^]^ Copyright 2009, Wiley‐VCH. H) Adapted with permission.^[^ ^90^ ^]^ Copyright 2010, Wiley‐VCH. I) Adapted with permission.^[^ ^47^ ^]^ Copyright 2015, Wiley‐VCH. J) Adapted with permission.^[^ ^103^ ^]^ Copyright 2013, American Chemical Society.

According to the interactions between nucleic acids and other materials, the assembly strategies of NAHs are dominant by physical crosslinking and chemical cross‐linking. A general scheme of DNA hydrogel assembly is shown in **Figure** **3**. Chemical cross‐linking is dominated by covalent bonds where the enhanced stability improves the mechanical strength, while the environmental stability of the hydrogel network prevents chemical bonds from breaking due to external factors. Therefore, chemical cross‐linking is considered permanent.^[^ ^41^ ^]^ Generally, DNA strands modified with functional groups are covalently immobilized onto synthetic polymers or biological macromolecules via polymerization reactions, coupling reactions, or enzymatic reactions^[^ ^74^, ^98^ ^]^ (Figure 3E,F). Comparatively, supramolecular DNA‐based hydrogels are mainly supported by noncovalent bonds, including hydrogen bonding, electrostatic interaction, and macrocyclic host‐guest interaction, and are equipped with greater flexibility, adaptability, and viscoelasticity.^[^ ^99^ ^]^

Most NAHs are stabilized and cooperate via multiple physical and chemical cross‐linking bridges. Nucleic acid amplification techniques, click reactions, and guest–host interactions are particularly popular in the building strategies of many soft biomaterials since the assembly process renders them more suitable for the incorporation of functional units, as well as their functional properties regarding efficiency, operability, and cost.

#### 2.3.1 Nucleic Acid Amplification Methods

The traditional methods used to obtain large amounts of raw materials for constructing NAHs primarily involve extraction kits or artificial synthesis. However, these approaches are limited regarding cost, purity, and synthetic technology and, therefore, cannot sustainably provide the support necessary for the development of smart NAHs. This provided a new direction for the application of nucleic acids amplification techniques. During the reasonable design of nucleic acid sequences, an appropriate nucleic acids amplification method or specific enzymes with extension functionality, such as terminal deoxynucleotidyl transferase (TdT), are selected for the highly efficient acquisition of substantial long DNA strand concentrations to serve as the backbone for DNA hydrogel construction (Figure 3F). Currently, three typical in vitro nucleic acid amplification techniques are used to produce hydrogel building blocks, which include the polymerase chain reaction (PCR), RCA, and the hybridization chain reaction (HCR).

**Figure 4:**
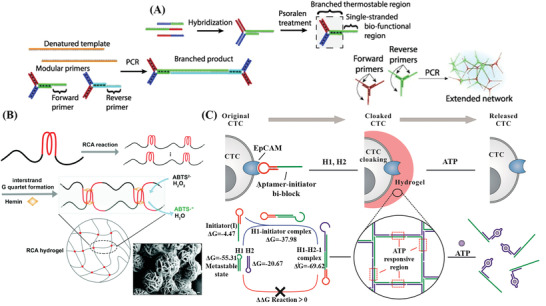
Nucleic acid amplification techniques for the construction of NAHs. A) Construction of DNA hydrogels based on thermostable branched DNA primers by PCR technology. Reproduced with permission.^[^ ^106^ ^]^ Copyright 2013, Wiley‐VCH. B) RCA hydrogels with stable catalytic activity for detection of glucose. Reproduced with permission.^[^ ^41^ ^]^ Copyright 2017, The Royal Society of Chemistry. Inset: SEM image of RCA nanoflowers (scale bars = 200 nm). Reproduced with permission.^[^ ^110^ ^]^ Copyright 2019, The Royal Society of Chemistry. C) Aptamer‐functionalized DNA hydrogels formed via HCR technology for live CTC analysis. Reproduced with permission.^[^ ^112^ ^]^ Copyright 2017, American Chemical Society.

PCR, a classic and representative technology for the amplification of DNA in vitro, along with molecular cloning and DNA sequence analysis methods, almost constitutes the basis of the entire modern field of molecular biology. Of these three experimental techniques, the PCR method first appeared in theory and is currently the most widely used in practice. The reaction mechanism of PCR, inspired by DNA semiconservative replication, is a process involving the extension of oligonucleotide primers under heat‐resistant DNA polymerase and the replication of genetic information along the template strand. After 20 to 30 cycles of being exposed to a process including high‐temperature denaturation, low‐temperature annealing, and moderate‐temperature extension, the target sequence can be amplified 10^6^‐fold.^[^ ^104^ ^]^ Additionally, the advantages of PCR include high specificity, sensitivity, and low demand for template purity. However, the bioanalytical PCR technique relies on sophisticated thermal cyclers, limiting its development for on‐site testing.^[^ ^104^ ^]^ Furthermore, the utilization of double‐strand DNA (dsDNA) building blocks generated via PCR cannot directly ligate with each other through complementary base pairing since the following products have blunt ends instead of sticky ends. However, with the advancement and development of PCR techniques, a blocker of oxyethylene glycol bridge was ingeniously inserted into the corresponding primers, generating sticky‐ended PCR products.^[^ ^105^ ^]^ Similar methods have been used for the fabrication of DNA hydrogels. Hartman et al.^[^ ^106^ ^]^ first reported a synthetic method for producing DNA hydrogels by using a thermostable branched DNA nanostructure with psoralen cross‐linking as modular primers (**Figure** **4**A). This PCR‐generated DNA hydrogel presented both self‐assembly and biological capabilities, allowing more sites to bind multiple customizable‐moieties, such as fluorescence dyes and genes, therefore, promoting the intelligence and functionality of NAHs.

RCA has been extensively exploited as a periodic nanoassembly technique for building DNA hydrogels that contain custom functionality and unique micromorphology. In principle, RCA is an isothermal amplification technology mediated by one or more single‐stranded circular DNA templates typically ranging from 13 to 240 nucleotides in length to catalyze and synthesize long stretches of DNA via DNA or RNA polymerase.^[^ ^107^, ^108^, ^109^ ^]^ Compared with PCR, the RCA technique, which has low operational requirements, eliminates a total dependence on a thermal cycler and thermostable DNA polymerases, while the process can be conducted in a solution, on a solid support, or in a complex biological environment.^[^ ^109^ ^]^ Furthermore, the amplification efficiency is adjustable by controlling the numbers of the template and the primer in the RCA reaction. Generally, linear RCA can be amplified up to 10^5^ times, while multi‐primed RCA can be amplified up to 10^9^ times.^[^ ^2^, ^104^ ^]^ Additionally, FNAs can be introduced into the tailor‐designed template sequence to reach the appropriate level of functionality. For example, Huang et al.^[^ ^41^ ^]^ designed circular cytosine‐rich DNA templates to produce long single‐strand DNA (ssDNA) containing multi‐repeated G‐rich sequences using RCA. Under proper conditions, these long ssDNA folds form intermolecular G4, providing enough cross‐linking force for the formation of hydrogels. The G4 structures contained within the DNA hydrogels were combined with hemin to produced stable HRP‐like catalytic functions for bioanalysis (Figure 4B). Moreover, on a microscopic level, RCA‐based DNA hydrogels exhibit a nanoflower‐like structure that holds great promise in drug delivery, in vivo imaging, tissue engineering, and 3D cell culture. The growth and change process of these nanoflower‐like structures have been monitored and recorded. Based on this information, Song et al.^[^ ^110^, ^111^ ^]^ further proved that the density and size of these nanoflower‐like structures could be adjusted by optimizing the RCA reaction conditions, building the relationship between the hydrogel state and the microcosmic morphology (inset of Figure 4B).

More recently, the linear and dendritic HCR, as the enzyme‐free nucleic acid‐activated chain reactions, have been extended to construct DNA hydrogels activated by DNA initiators. Generally, HCR triggers two hairpins with sticky ends via ssDNA as an initiator, successively exposing new single‐stranded regions until the hairpins are fully opened to complete DNA self‐assembly, finally obtaining DNA polymers with multiple repeating units.^[^ ^109^ ^]^ The construction of the HCR‐based DNA hydrogels is achieved via two strategies. One is to insert FNAs into two hairpin structures before HCR for the subsequent bioanalysis. Song et al.^[^ ^112^ ^]^ created an aptamer‐triggered HCR‐based DNA hydrogel for in situ identification and direct capture of the active circulating tumor cells (Figure 4C). The other method involves cross‐linking different FNA units or nanoparticles with the double‐helix product during or after HCR. For example, large amounts of ssDNA, as the initiator produced by a Hg (II)‐responsive exponential amplification reaction (EXPAR), triggered the dendritic HCR to form the DNA hydrogel network, trapping and aggregating AuNPs to generate the detection signal.^[^ ^113^ ^]^ Besides, the hybridization exchange as a special HCR can achieve similar cascaded amplification to produce a long DNA duplex. Cangialosi et al.^[^ ^114^ ^]^ developed a shape‐change DNA‐polyacrylamide hydrogel by using hybridization exchange with well‐designed DNA sequences to substantially prolong the cross‐links and further induce hydrogel to 100‐fold volumetric highly swelling.

Furthermore, the TdT‐catalyzed DNA synthesis technique was introduced to generate essential building blocks for the development of NAHs. TdT can catalyze the addition of dNTP to the 3’‐OH ends of oligo‐ and polynucleotide primers without being directed by a template. By extending TdT in four directions, Binbin et al.^[^ ^39^ ^]^ prepared the X‐shaped DNA units with poly‐A and poly‐T tails, which hybridized with each other to assemble and form the hydrogel. This study confirmed that the utilization of TdT was efficient in decreasing the consumption of DNA units while improving the mechanical strength of the hydrogels via the hybridization of the poly‐A and poly‐T tails.

#### 2.3.2 Click Chemistry and Bioorthogonal Chemistry

Click chemistry has attracted increasing attention since it was first proposed by chemists Sharpless, Kolb, and Finn in 2001. Its purpose is to explore a series of powerful chemical reactions, while displaying characteristics, such as high reliability, selectivity, and modularization, as well as exhibiting the ability to rapidly synthesize both useful new compounds and combinatorial chemical libraries via the binding of carbon‐heteroatom bonds (C‐X‐C).^[^ ^127^ ^]^ The click reactions are identified by their main characteristics, which include the following: the starting materials and reagents for the reaction are easily obtained; the reaction conditions are simple, mild, and insensitive to oxygen and water; the occurrence of the reaction relies on a high thermodynamic driving force (> 20 kcal mol^‐1^), and only harmless by‐products are produced or can be removed by non‐chromatographic methods.^[^ ^115^ ^]^ With these features as the judging criteria, click reactions roughly cover four categories of addition reactions, which include cycloaddition reactions, such as the 1,3‐dipole‐cycloaddition reaction (CuAAC) and the Diels‐Alder Reaction; nucleophilic ring‐opening reactions, such as those involving epoxides, aziridines, and cyclic sulfates; carbonylation reactions, such as the formation of oxime ethers, hydrazones, and aromatic heterocycles; and carbon‐carbon multiple bond addition reactions, denoting epoxidation, dihydroxylation, and azide–phosphine coupling reactions, such as Staudinger ligation.^[^ ^119^ ^]^ Click chemistry has almost permeated through all aspects of modern chemistry and biology. Remarkably, several highly efficient click reactions have been applied for molecular self‐assembly, surface functionalization of materials, and immobilization of biomolecules in biomaterials science, especially the studies of smart NAHs. Thus, click chemistry provides the innovative cross‐linking approach and functionalized strategy for DNA chains and other materials composed of NAHs.

However, the application of partial click reactions still presents challenges in vivo and the intracellular environment. For example, abundant experimental data have shown that CuAAC, as the most widely‐used click reaction, is not suitable for labeling biomolecules in biological systems due to the potential cytotoxicity of copper (I).^[^ ^120^, ^121^, ^122^, ^123^, ^124^, ^125^, ^126^ ^]^ In fact, the metal catalyst is difficult to eliminate from the system, and excessive intake causes side effects, such as hepatitis, Alzheimer's disease, and neurological disorders.^[^ ^116^ ^]^ Consequently, a new concept, namely bioorthogonal chemistry, was presented by Professor Bertozzi and colleagues based on the gradually‐developing click chemistry. This approach refers to the chemical reactions performed in the living system without interfering with its biochemical processes.^[^ ^127^ ^]^ Such reactions should be equipped with the following three elements: the reaction must occur in an aqueous solution; the catalyst or reactant participating in the reaction must be nontoxic; two functional groups of reactants must not exist in the biological system, while cross‐reactions should not occur with any of the functional groups in the biological system. Moreover, the bioorthogonal reactions display the same characteristics as click reactions, including the rapid reaction rate, high yield, the absence of excess by‐products, excellent biocompatibility, and easy access to reaction starting materials.^[^ ^116^ ^]^ The increased research popularity of biological systems has focused the attention on metal‐free click reactions, as members of the bioorthogonal reactions, and utilizing them for fabricating NAHs and other biomaterials, such as the Diels–Alder reaction,^[^ ^128^ ^]^ strain‐promoted azide–alkyne cycloaddition (SPAAC),^[^ ^98^ ^]^ and thiol–ene radical addition.^[^ ^67^ ^]^ Therefore, the concept of bioorthogonality can be deemed an extension of click chemistry, while its application fields include a broad spectrum of self‐assembling biomaterials in various complex biological systems.^[^ ^129^ ^]^

#### 2.3.3 Macrocyclic Host–Guest Interactions

Macrocyclic host–guest interactions, as noncovalent cross‐linking forces, have been widely used to synthesize multifarious biomolecule‐based supramolecular hydrogels but had not been utilized to produce NAHs until recently.^[^ ^78^, ^98^, ^130^ ^]^ Given their excellent dynamic nature, the host–guest interactions, provided by low molecular weight gelators or physically cross‐linked polymers, drive the self‐assembly of DNA strands with each other, or DNA molecules with other polymers, to form DNA‐based supramolecular hydrogels with outstanding chemical and physical properties, such as shape‐memory, self‐healing, and swelling.^[^ ^98^, ^131^, ^132^, ^133^, ^134^ ^]^ In supramolecular hydrogels, the multiple water‐soluble macrocyclic hosts have been introduced into the building blocks or scaffolds, such as cyclodextrins (CD), cucurbit[n]urils (CB[n]), calix[n]arenes, and pillar[n]arenes (PA[n]s). Notably, due to their intrinsic water solubility, both CD and CB[n] easily react with hydrophilic DNA molecules and specifically bind with the complimentary guest molecules in aqueous media.^[^ ^135^, ^136^ ^]^ The binding behavior between the macrocyclic hosts and their complementary guests exhibit high specificity, binding‐strength adjustability, and dynamic property, providing excellent opportunities for designing or customizing the functions of DNA‐based supramolecular hydrogels.^[^ ^130^ ^]^ Generally, the construction of smart NAHs via mediated macrocyclic host‐guest interactions primarily follow three approaches: (a) linking the macrocyclic host to the polymer chain, (b) linking the guest group to the polymer chain, or (c) connecting both the host and guest to the polymer chain.^[^ ^130^ ^]^ Li et al. designed a supramolecular double‐network hydrogel combined with physical interpenetration, with two individual self‐assembly hydrogel systems based on DNA hybridization and macrocyclic host‐guest interactions.^[^ ^137^ ^]^ Even though these two hydrogel networks had no crosslinking interactions with each other, after merging, the entire dual‐hydrogel system displayed enhanced strength with excellent elasticity, ductility, shear‐thinning, and thixotropy. Additionally, different specific binding sites were embedded in each hydrogel scaffold of this novel soft material, allowing it to selectively achieve enzymatical responsiveness to nuclease, cellulase, and small molecules. It is predicted that smart NAHs, dominated by macrocyclic host‐guest interactions, show promise for application in molecular identification, drug delivery, and tissue engineering.

## 3 The Functional Features and Smart Strategies of NAHs

NAHs show significant promise, both in vivo and in vitro, due to their similarity to natural tissues and their ability to mimic biomechanical processes.^[^ ^19^, ^138^ ^]^ The unique features of these hydrogels can be ascribed to two aspects of nucleic acids. The first involves high biocompatibility, stability, precise programmability, conformation flexibility, as well as facile synthesis and modification, while the second denotes the mechanical properties of the hydrogel material, such as elasticity, extensibility, and stiffness.^[^ ^5^, ^19^ ^]^ Furthermore, FNAs and polymers, as the primary components of smart NAHs, not only help to enhance their basic biophysical characteristics but also provide additional bioactivity and smart strategies for the expansion of their application ranges. Therefore, examining the functional features and smart strategies provide essential guidance for the design and fabrication of smart NAHs in practical applications.

### 3.1 The Basic Biophysical Features of NAHs

The basic biophysical features of smart NAHs mainly include two aspects: the essential features of nucleic acids, such as target specificity, biocompatibility, and stability, as well as the mechanical properties and permeability provided by the microstructure of the hydrogel matrix.

i) Biocompatibility: Biocompatibility is essential for biomaterials applied in vivo. For DNA‐based hydrogels, especially pure DNA hydrogel, the raw materials obtained by the extraction of natural organisms or artificial synthesis are low in toxicity and harmless, hardly causing inflammation. Based on the excellent biocompatibility and degradability of DNA itself, the DNA hydrogels can be used for cell proliferation and migration in tissue engineering, since they are controllable for the slow release of drugs over extended periods with good degradation rates.^[^ ^12^, ^139^ ^]^

ii) Stability: Generally, DNA molecules retain enhanced stability when subjected to intense heat, pressure, and chemical processing.^[^ ^140^ ^]^ Except for critical factors, such as the proportion of four nucleotides and the hydrogen bonds formed between the base pairs, the stability of DNA‐based hydrogel networks exhibits a close relationship with the length and concentration of the DNA building blocks and their various interactions. The incorporation of G4, i‐motif, or other FNAs with specific spatial structures, into DNA hydrogels, further elevates the degree of cross‐linking and the stability of the hydrogel networks.^[^ ^18^ ^]^ Additionally, the interaction between DNA and other polymers or nanoparticles also provides support for the stability of the hydrogels.

iii) Synthesis and modification: Modern DNA synthesis techniques allow for the rapid preparation of large quantities of DNA via chemical synthesis dominated by the automated solid‐phase technique, as well as enzymatic synthesis dominated by PCR amplification techniques.^[^ ^5^, ^6^, ^7^ ^]^ Additionally, DNA can be effortlessly modified with multifarious functional groups such as acrydite, amino, and thiol, as well as small molecules, such as biotin, ferrocene, and FAM.^[^ ^5^ ^]^ Nucleic base modification can easily be achieved using a DNA synthesizer, providing an excellent foundation for assembling functional blocks in the NAHs. iv) Mechanical properties: DNA chains with different levels of rigidity and flexibility provide DNA hydrogels with elasticity, viscosity, elasticity, and other mechanical properties, which are determined and regulated by the assembly conditions, such as the concentration, length, and flexibility of the DNA chains, as well as the proportion of the components.^[^ ^35^, ^141^ ^]^ Furthermore, ssDNA, at a consistent length of about 2 nm, is more flexible than dsDNA at approximately 50 nm, while bending or twisting dsDNA expends about 50 times as much energy as ssDNA.^[^ ^142^, ^143^, ^144^, ^145^ ^]^ Moreover, different DNA nanostructures formed by FNAs under specific conditions also have a significant influence on the mechanical properties of hydrogels. Consequently, the selection of conventional ssDNA, dsDNA, and unique DNA nanostructures allows the mechanical strength of the DNA‐based hydrogels to be adjusted for different applications, such as drug delivery and tissue engineering.^[^ ^146^ ^]^ v) Permeability: Due to their porous structure, NAHs generally exhibit high permeability which is, closely associated with the porosity, density, and degree of cross‐linking of the hydrogel network, as well as with the effects of controlled drug release. The delivery and release of drugs are generally understood via diffusion‐controlled mechanisms, while its rates depend on the diffusion coefficient.^[^ ^147^ ^]^ The ideal controlled drug release can be achieved by decreasing the diffusion coefficient, such as reducing the mesh size or enhancing the interaction between the drugs and the gel matrix.^[^ ^148^ ^]^ The reality is that permeability, as a special behavior of diffusion, has become a double‐edged sword in hydrogel applications. The high permeability of hydrogels leads to the rapid release of loaded cargo, which not only decreases the biological efficacy of the released substances but may also cause severe side effects in vivo.^[^ ^149^ ^]^ However, the high permeability can also facilitate sufficient contact with specific stimuli, permitting a rapid response in the smart DNA hydrogels.^[^ ^92^ ^]^ Consequently, a technique was used by which a specific substance was added to induce a slight change in the microstructure of the hydrogel, adjusting its permeability to prepare a DNA hydrogel film, which was associated with the capillary behavior for the ultra‐trace detection of cocaine.^[^ ^150^ ^]^

### 3.2 The Abundant Bioactivities of NAHs

One of the important reasons that NAHs have attracted particular interest from researchers is that their abundant bioactivities are continuously explored and introduced into the preparation of hydrogels to develop function‐rich soft biomaterials. A large part of these remarkable bioactivities originates from FNAs or other compounds with special features.

i) Target specificity: DNA‐based hydrogels can be programmed by precisely designing DNA sequences to carry specific genetic information or to display identification functions. In most studies, different FNAs with excellent molecular recognition properties, such as aptamers and DNAzymes are introduced into DNA hydrogels, allowing the reaction sites to identify the target or act as tools for target genes or drug delivery.^[^ ^37^ ^]^

ii) Biocatalytic activity: The biocatalytic activity of NAHs is realized via HRP mimicking DNAzymes or metal‐dependent nicking DNAzymes. The former relies on the G4 structure that binds with hemin in the presence of K^+^, Na^+^, or other specific metal ions to form HRP mimicking DNAzymes that can catalyze the oxidation of many substrates via hydrogen peroxide (H_2_O_2_) to generate colored products. These products can act as a signal readout to indicate the progression of the catalytic reaction and can provide visual prompts for assessment.^[^ ^41^, ^63^, ^103^, ^151^, ^152^, ^153^, ^154^ ^]^ The commonly used substrates include 2,2’‐Azino‐bis (3‐ethylbenzothiazoline‐6‐sulfonic acid) diammonium salt (ABTS^2−^), 3,3’,5,5’‐tetramethylbenzidine (TMB), dopamine, and 10‐acetyl‐3,7‐dihydroxyphenoxazine (Amplex Red, AR).^[^ ^153^ ^]^ Additionally, various nanomaterials, such as AuNPs, Fe_3_O_4_ nanoparticles, CeO_2_ nanoparticles, V_2_O_5_ nanowires, Co_3_O_4_ nanoparticles, and TiO_2_ nanotubes have exhibited the same enzyme‐mimicking biocatalytic activity as G4, occurring in similar catalytic reactions,^[^ ^154^ ^]^ and encoding the specific sequences of metal‐dependent nicking DNAzymes into DNA strands to synthesize DNAzyme‐based hydrogels. Generally, the ends of a substrate DNA strand are labeled with a dye/fluorophore and a quencher, respectively. In the presence of cofactor metal ions, the substrate DNA strand containing an RNA base is split from the cleavage site. The labeled ends of the substrate generate corresponding responsive signals to indicate the catalytic processes.^[^ ^63^, ^155^ ^]^

iii) Antibacterial activity: NAHs provide an appropriate matrix to hold the photosensitizer or microbicide for better retention and more potent antimicrobial action. These small molecules displaying antibacterial activity can chemically cross‐link DNA either through covalent bonds or physically via noncovalent bonds to form the NAHs, while their antibacterial mechanisms are achieved through the permeability of the hydrogel material or by stimuli‐responsive release.^[^ ^7^, ^156^ ^]^

### 3.3 The Stimuli‐Responsive Properties of Smart NAHs

During the development process of NAHs, their responsiveness is explored and widely used to enhance their functionality, playing an essential role in the development of smart NAHs. Stimuli‐responsive DNA‐based hydrogels represent a broad class of hydrogels that are responsive to external stimuli and undergo switchable gel‐to‐solution or gel‐to‐solid transitions, which can be monitored according to the visual changes in color or fluoresce.^[^ ^2^, ^157^ ^]^ The most commonly‐applied triggers primarily include temperature, pH, light, metal ions, enzymes, and small molecules.^[^ ^30^, ^158^, ^159^, ^160^ ^]^ Recently, new stimuli, such as redox,^[^ ^71^, ^161^ ^]^ magnetic fields,^[^ ^9^, ^10^, ^84^ ^]^ electric fields,^[^ ^159^ ^]^ and near‐infrared radiation^[^ ^162^ ^]^ are gradually being used to regulate the properties of hydrogels.^[^ ^2^ ^]^ In smart DNA‐based hydrogels, these stimuli are provided by FNAs, small molecules, and polymers with unique features. For example, Guo et al.^[^ ^62^ ^]^ designed a temperature‐responsive DNA/pNIPAM hydrogel based on the i‐motifs formed by the copolymerization of cytosine‐rich ssDNA modified with AM and NIPAM. With a temperature change, the DNA/pNIPAM hydrogels underwent a reversible transformation between a gel and a solid‐state. When the temperature increased to 45 °C, the hydrogel transformed into a solid‐state with the dissociation of the network. When the temperature dropped to 25 °C, it reverted to a swollen hydrogel with the reformation of the network. Hu et al.^[^ ^48^ ^]^ assembled a pH‐responsive AM‐DNA hydrogel using two triple‐helix DNA bridging units, namely T‐A•T and C‐G•C^+^ base triplets. At pH 7.0, the formed hydrogel was simply supported by the T‐A•T triple helix DNA bridging unit. At pH 10.0 and pH 5.0, the transition of the hydrogel from a gel state to a quasi‐liquid state occurred with the dissociation of the T‐A•T triple helix DNA bridging unit and the reformation of the C‐G•C^+^ triple helix DNA bridging unit, respectively. According to this mechanism, the phase transformation of the pH‐responsive AM‐DNA hydrogel was achieved in three different pH conditions. Lee et al.^[^ ^163^ ^]^ constructed a water‐responsive DNA hydrogel via hyperbranched RCA with multiple designed primers. The subsequent DNA hydrogel exhibited unusual metaproperties since the hydrogel changed to a liquid‐like state exhibiting fluidity by removing the water from the physical environment, while it rapidly recovered to its original solid‐state in a water‐filled environment. Therefore, based on its unique molecular and environmental response properties, stimuli‐responsive DNA‐based hydrogels exhibit broad application prospects in biosensing, bioimaging, drug delivery, tissue engineering, and novel soft materials, laying the foundation for its intellectualization by incorporating it with stimuli in applications.

### 3.4 The Shape‐Memory and Self‐Healing Properties of Smart NAHs

In recent studies, several stimuli‐responsive DNA‐based hydrogels with reversible properties have been developed, and their various functions were achieved with phase transitions and stiffness changes to the hydrogel network, which was initiated by simultaneous responses to single or diverse external stimuli.^[^ ^7^ ^]^ Within the broad interest in smart polymers, DNA‐based hydrogels with shape‐memory and self‐healing properties are considered as the next smart pathway for the application of stimuli‐responsive soft biomaterials.^[^ ^58^, ^164^, ^165^, ^166^, ^167^ ^]^

The shape‐memory properties of DNA‐based hydrogels are achieved during the fabrication of the original foundation of the permanently‐shaped structure. In the presence of an auxiliary trigger, this structure transitions into a temporarily‐shapeless structure programmed with “memory codes” that can be restored to the original shape by the initiation of appropriate counter stimuli.^[^ ^72^, ^168^, ^169^ ^]^ Therefore, it is evident that two basic features determine the realization of the shape‐memory properties: one denotes the stimuli‐responsive cyclic phase transitions of the hydrogel system, while the other represents the substantial structural and functional encoded information designed as “memory codes”.^[^ ^6^, ^7^, ^8^, ^9^ ^]^ Diverse, functional cross‐linking units are incorporated to provide the assembly of the DNA‐based hydrogels with shape‐memory properties, such as duplex/pH‐responsive FNA units (e.g., i‐motif or triplexes with T‐A•T or C‐G•C^+^ base triplets),^[^ ^46^, ^138^, ^159^, ^169^, ^170^ ^]^ K^+^/crown ether formation and separation of duplex/G4,^[^ ^146^, ^159^ ^]^ strand displacement in the presence of appropriate nucleic acid strands,^[^ ^168^ ^]^ and photoisomerization of trans/cis‐azobenzene tethered to the nucleic acids.^[^ ^58^, ^171^, ^172^ ^]^ For example, The K^+^/CE responsive AM‐DNA hydrogel was assembled by becoming part of the G4 and double‐helix structures. The addition of CE into the hydrogel system allowed the formation of a hydrogel with stereotypical structures by destroying G4. When K^+^ was added, the hydrogel returned to an amorphous structure with the reformation of G4.^[^ ^14^ ^]^ The extensive applications currently suggested for shape‐memory DNA‐based hydrogels as smart, soft biomaterials include controlled drug delivery and release,^[^ ^46^ ^]^ switchable catalysis,^[^ ^58^, ^142^ ^]^ and controlled transport.^[^ ^35^, ^173^ ^]^

Similarly, the self‐healing ability of DNA‐based hydrogels is another related functional feature derived from stimuli‐responsiveness. A self‐healable hydrogel must be equipped with the ability to regenerate multiple new bonds by harnessing the original components of the existing structure at the damaged or cleaved region.^[^ ^174^ ^]^ Generally, the self‐healing DNA‐based hydrogels are dominated and stabilized by cooperative bridges. The damaged soft DNA hydrogel in a quasi‐liquid state is conjugated by one building unit and is further restored to an intact hydrogel by triggering the second building unit used for enhancing the stiffness.^[^ ^58^, ^174^ ^]^ Although the triggers that induce the self‐healing process are the same as those commonly used in the stimuli‐responsive system, most of the functional bridges are established based on dynamic supramolecular cross‐linking, such as ligand‐receptor interactions,^[^ ^175^, ^176^ ^]^ covalent acylhydrazone bonds,^[^ ^128^ ^]^ hydrogen bonds of DNA hybridization,^[^ ^15^, ^94^, ^177^ ^]^ *π*‐*π* stacking,^[^ ^76^ ^]^ and hydrophobic interactions.^[^ ^178^ ^]^ A variety of DNA‐based hydrogels with the self‐healing capability for bio‐adhesion, tissue engineering, and tissue recovery were suggested, and some of them have manifested an intelligent tendency, such as a supramolecular polypeptide–DNA hydrogel applied for in situ 3D multilayer bioprinting.^[^ ^15^, ^178^, ^179^ ^]^

## 4 Applications of Smart and Functionalized NAHs

As a promising biomaterial, the application development of NAHs always conforms to two general directions: bioanalysis and biomedicine. At present, various types of NAHs have been actively explored, presenting application potential in multiple subfields of these two directions, such as immunomodulation, food safety, drug delivery, biomimetic simulation, and tissue engineering. Especially in recent years, the change in market demand and advances in research quality speed have increased the development of smart and multi‐FNA‐based hydrogels that exhibit diverse functions and properties. Compared with early DNA hydrogels that only possessed a single function or were aimed at a single target, these promising soft biomaterials provide high‐tech integration systems. These systems combine novel techniques, innovative materials, and portable devices to provide irreplaceable performance in a broad range of experimental research to satisfy further mass production, high speed, high throughput, high specificity, high sensitivity, and other demands, significantly contributing to the application expansion to new frontiers, such as cell culture, 3D bioprinting, and treatment and analysis of environmental pollutants. This section highlights typical smart and multifunctional applications of NAHs in the conventional and new subfields.

### 4.1 NAHs for Bioanalysis

#### 4.1.1 Biosensing

Due to the remarkable advantages of transforming the traditional liquid‐phase reaction system into a 3D network structure to improve the loading capacity of analytes, the NAHs have been designed as a series of innovative biosensors with outstanding features of portability, repeatability, high sensitivity, and high selectivity. Therefore, they are developed for the real‐time monitoring of biochemical processes and the qualitative or quantitative analysis of diverse analytes in vitro and in vivo, particularly displaying extensive application prospects in clinical diagnosis, point‐of‐care testing (POCT), personalized healthcare, environmental monitoring, and food safety. Generally, the smart design strategy of NAHs for biosensing denotes a combination of synthetic polymers, long ssDNA, or branched dsDNA as a scaffold structure, and various FNA units as cross‐linkers.^[^ ^180^, ^181^ ^]^ Once the analytes are directly and specifically recognized by the corresponding structure units of the hydrogel network, or indirectly stimulate the change in environmental factors, the hydrogel system provides swift physical responses to the swelling behavior, phase state, mechanical strength, or the release of cargo encapsulated in advance to reflect the presence and quantity of the target. These physical changes are subsequently converted to readable signals for the detection and analysis of the targets.^[^ ^180^, ^181^, ^182^ ^]^ The NAHs, as special media, or sites for biosensing, not only transform the traditional liquid‐phase reaction system into a 3D network structure to improve the loading capacity of analytes but also convert the analytes into easy‐to‐process sensing signals for easy detection.^[^ ^158^, ^183^ ^]^ To date, the NAHs applied for biosensing involved a wide range of analytes, mainly including ions, living cells, proteins, viruses, bacteria, and toxins.^[^ ^184^ ^]^

During the early part of the twenty‐first century, researchers focused on the intensive development of FNA‐based hydrogels with a simple design for the detection of a specific target, while a small number of them have revealed a promising trend in future smart development. For example, the team of Tan et al. incorporated the research experience with aptamers into the development of molecular detection platforms and molecular logic gates based on aptamer‐cross‐linked hydrogels for bioanalysis.^[^ ^90^, ^185^ ^]^ Two detection platforms based on AND and OR logic gate systems rapidly responded to logic simulation with cocaine and ATP as the targets via a transition of gel to a solution to indicate the logic analysis process visually, resulting in the dissociation of the hydrogel and the controlled release of BSA‐modified AuNPs from the hydrogel network. In the meanwhile, analogs of cocaine and ATP were used to evaluate the selectivity of this detection system and the results showed that all the analogs did not cause the color change. In other words, the aptamers of cocaine and ATP with high selectivity cannot associate with these analogs to launch the detection system. This established logic detection platform combined with a simple stimuli‐responsive design and aptamer‐cross‐linked hydrogels achieved FNA engineering development and application in biosensing.^[^ ^185^ ^]^ Then, the concentrated development of different stimuli‐responsive DNA hydrogels provided a series of innovative biomaterials for utilization in biosensing. Willner et al. developed several smart DNA‐polymer hydrogels cross‐linked by a succession of different FNA subunits, such as the catalytic label hemin/G4,^[^ ^63^ ^]^ cytosine‐Ag^+^‐cytosine complexes,^[^ ^186^ ^]^ i‐motifs,^[^ ^62^, ^169^ ^]^ and Hoogsteen triplex structures.^[^ ^46^ ^]^ The gel/solution transitions of these DNA hydrogels, adjusted simultaneously by one or more stimuli of specific active molecules, pH, or metal ions support, establish a broader range of targets for detection and provide more signal output choices. Consequently, these successfully‐developed biomaterials pave the way for the construction of novel hydrogel biosensor systems. Currently, smart NAHs for biosensing exhibit a multi‐angle and multi‐level dynamic development trend in synthetic techniques, advanced materials, and pluralistic analytes.

**Figure 5:**
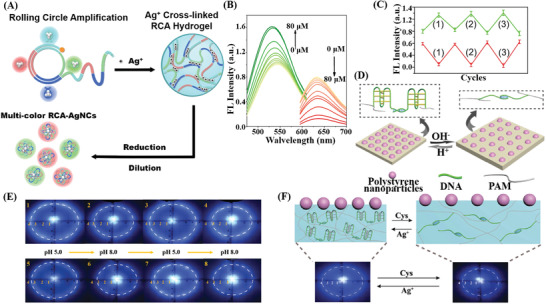
Integration of smart DNA hydrogels with amplification technology and novel nanomaterials for biosensing. A–C) Scheme of an RCA‐AgNCs hydrogel and its application of fluorescent biosensing for ROS/RNS detection. Reproduced with permission.^[^ ^187^ ^]^ Copyright 2018, American Chemical Society. D) Scheme of pH‐responsive 2D PC‐DNA hydrogel film, E) and its reversible changes between hydrogel state (1/3/5/7) and the pre‐gel state (2/4/6/8) reflected by Debye diffraction ring. F) Illustration of Ag^+^/Cys‐responsive 2D PC‐DNA hydrogel film. Reproduced with permission.^[^ ^188^ ^]^ Copyright 2019, Wiley‐VCH.

In recent years, the superiority of acid‐based hydrogels has been significantly enhanced by combining them with new application modes of nonspecialized technology or with newly explored interdisciplinary findings, especially nucleic acid amplification technology, and PC. DNA hydrogels based on RCA technology have been applied for biological sensing due to the easy acquisition of a considerable number of identification sites to realize signal amplification and generate a unique morphological microstructure. Li et al.^[^ ^187^ ^]^ quantitatively synthesized the RCA‐stabilized multicolor silver nanoclusters (AgNCs) in situ in a DNA hydrogel consisting of cross‐linked enzymatically amplified polymeric DNA with cytosine‐rich sequences in the presence of Ag^+^. A potential fluorescent probe was created for the real‐time monitoring of the •OH levels to reflect the reactive oxygen/nitrogen species (ROS/RNS) in live cells. The red‐emitting species could be converted to green‐emitting species simply under strong oxidation conditions, which was conducive to excellent selectivity for the detection of ROS/RNS (**Figure** **5**A,B). Additionally, the intelligence of this sensing system was confirmed since the fluorescence changes could be quickly reversed by adding a strong reducing agent, NaBH_4_, leading to an increase in the red emission and a decrease in the green emission, allowing them to return to their initial states. These reversible interconversions between the red and green emissive species can repeatedly respond to the redox switches for several cycles in the biomedical applications of high‐efficient intracellular sensing (Figure 5C). Besides the expansion of RCA technology into DNA hydrogels, the research on PC is flourishing and has now permeated molecular detection by being incorporated into DNA hydrogels. Wang et al.^[^ ^188^ ^]^ embedded the i‐motif and self‐complementary structures to construct a shape‐memory PC‐DNA hydrogel film in which shape changes were triggered by changes of external pH stimuli, modulating the lattice spacing of the 2D PC array on the surface of the film. This reversible process reflected the diameter changes in the Debye diffraction ring, which can be observed and recorded as signal output for bioanalysis (Figure 5D,E). Based on the universal working principle, the regulation of 2D PC‐DNA hydrogel film still performed well after replacing pH with Ag^+^/Cys stimuli (Figure 5F). In the verification experiment of selectivity, even though 2D PC–DNA hydrogel film was treated by using other metal ions with higher concentrations as control, the results showed no any obvious effects on spacing and verified good selectivity of DNA hydrogel film. Therefore, it is apparent that DNA hydrogels are highly inclusive and adaptable to the establishment of biosensors.

**Figure 6:**
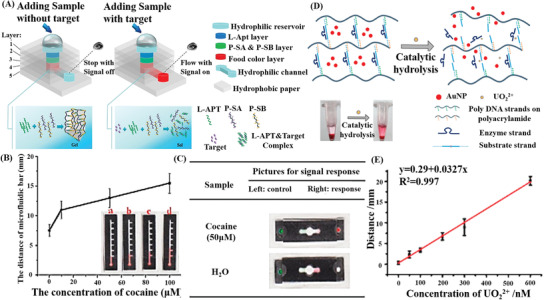
Examples of portable smart and functionalized DNA‐hydrogel biosensors for on‐site detection or POCT. A) Schematic illustration of simulated structures of the μPAD visual biosensor. B,C) and its application for cocaine qualitative detection. Reproduced with permission.^[^ ^191^ ^]^ Copyright 2015, American Chemical Society. D,E) The working principle of AuNPs mediated portable visual biosensing V‐chip integrating UO_2_^2+^‐responsive hydrogel and its bioanalysis results. Reproduced with permission.^[^ ^193^ ^]^ Copyright 2016, Elsevier B.V.

Furthermore, in the newly‐developed biosensors applied for on‐site detection or POCT, many novel nanomaterials or portable devices have been combined with hybrid NAH systems to improve the ease of operation, dynamic response speed, signal‐reading modes, detection sensitivity, and other properties of biosensors. Yang et al. designed a succession of typical portable sensors integrating different target‐responsive DNA hydrogels with glucometers,^[^ ^189^ ^]^ volumetric bar‐chart chips (V‐Chip),^[^ ^190^ ^]^ microfluidic paper‐based analytic devices (μPAD),^[^ ^191^ ^]^ pressure meters,^[^ ^192^ ^]^ capillary tubes,^[^ ^150^ ^]^ or other general portable readout devices for the simple, sensitive, and user‐friendly detection of various targets. Remarkably, the miniaturization and integration of a micro‐total analytical system are becoming mainstream to encourage the development of POCT. Wei et al.^[^ ^191^ ^]^ established a versatile point‐of‐care assay platform for the simultaneous detection of multiple targets based on a μPAD using a target‐responsive hydrogel to mediate the fluidic flow and signal readout. The aptamer‐cross‐linked hydrogel, as a target‐responsive flow regulator, was formed in the absence of a target to stop the flow in the μPAD flow channel. Contrarily, the preferential interaction between the introduced target and aptamer impeded the formation of the hydrogel, allowing free fluidic flow to carry the indicator to the observation spot and produce a “signal on” readout (**Figure** **6**A). This assay platform, based on the μPAD, can achieve high‐throughput detection, and visualize the testing results within 6 min without the aid of sophisticated instrumentation (Figure 6B,C). In the meanwhile, the selectivity test results suggested that a red color appeared only when the concentration of cocaine reached as high as 50 µM. It was evident that the hydrogel‐based μPAD is also applicable for the real sample analysis, even in the complex biological matrix, such as urine. Similarly, Yishun et al.^[^ ^193^ ^]^ designed a uranyl ion (UO_2_^2+^) responsive, smart DNA‐grafted PAM hydrogel, cross‐linked with the substrate strand and enzyme strand of the UO_2_^2+^‐dependent DNAzymes. This hydrogel‐based biosensor was used for colorimetric analysis by encapsulating AuNPs in the DNAzyme cross‐linked hydrogel to indicate the concentration of UO_2_^2+^ (Figure 6D). The detection limit of the UO_2_^2+^ was as low as 100 × 10^‐9^ m, which was observable with the naked eye (Figure 6E). The DNAzyme‐based hydrogel detection system also displayed high selectivity in the presence of other cation ions with 100‐fold higher concentrations. Compared with the previous example, the most apparent difference was that this smart biosensor used a previously developed V‐Chip for the quantitative detection of UO_2_^2+^ in water by encapsulating Au–Pt nanoparticles in the hydrogel to avoid the visual errors caused by naked eye observation, but lacked high‐throughput detection capability.

**Figure 7:**
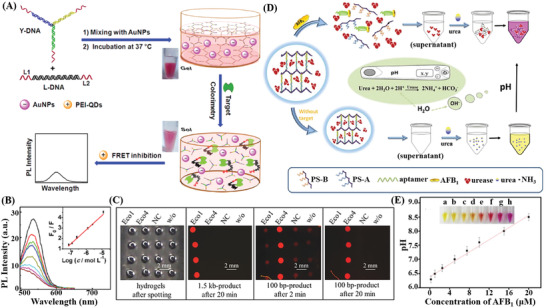
Design principle and detection effects of smart and functionalized DNA‐hydrogel biosensors for different analytes. A,B) Scheme of hydrogel‐based dual‐signal aptasensor for the colorimetric and fluorescent detection of thrombin, and its fluorescent detection results. Reproduced with permission.^[^ ^75^ ^]^ Copyright 2013, American Chemical Society. C) Detection process of PCR‐assisted hydrogel array fluorescent biosensor with high specificity.Reproduced with permission.^[^ ^194^ ^]^ Copyright 2016, Wiley‐VCH. D,E) The principle and results of pH meter embedded with pH‐responsive hydrogel‐based aptasensor for AFB1 visual detection. Reproduced with permission.^[^ ^195^ ^]^ Copyright 2017, Elsevier B.V.

The smart and functionalized assays conducted by biosensors containing NAHs have been expanded for different types of analytes in vitro or in vivo. In addition to well‐studied miRNA and some heavy metal ions, the hydrogel‐based biosensors have been designed specifically for protein,^[^ ^75^ ^]^ bacteria,^[^ ^194^ ^]^ and biotoxin^[^ ^194^, ^195^, ^196^ ^]^ detection. In a study by Zhang et al.,^[^ ^75^ ^]^ Y‐shaped DNA and an aptamer linker were self‐assembled to fabricate a pure DNA hydrogel, entrapping AuNPs as indicator agents for the subsequent visual detection (**Figure** **7**A). The detection system underwent a gel‐to‐sol transition in the presence of thrombin as the model analyte, leading to the release of negatively charged AuNPs, gradually approaching the positively charged, self‐synthesized PEI‐QDs. The electrostatic interaction between PEI‐QDs and AuNPs caused a fluorescence resonance energy transfer, finally quenching the fluorescence signal of the PEI‐QDs to achieve the sensitive detection of thrombin (Figure 7B). The target‐responsive, pure DNA hydrogel dismissed synthetic polymers as the scaffold, further improving the biocompatibility of this biosensor, and providing a universal switchable material for signal transduction. With the increasing demands regarding food safety evaluation and risk analysis, DNA hydrogels in various forms have been applied for the rapid detection of food contaminants, while performing different functions in biosensors. Beyer et al.^[^ ^194^ ^]^ coupled arrayed 3D hydrogel decorated chips with the PCR technique to amplify the target gene for bacterial detection. The hydrogel array, which consisting of 3D hydrogel droplets with high gel porosity and polymerized under visible light, provided a larger surface area than 2D surfaces for functionalization, further promoting the accessibility of gel‐immobilized DNA molecules (Figure 7C). Without the need for prior template DNA extraction, this PCR reaction was facilitated via direct chip implementation, with the bacterial lysate containing the cellular debris, while the accumulated specific fluorescence hybridization signals were immediately detectable via microscopy after undergoing the cycles of denaturation, annealing, and extension. Furthermore, the addition of portable devices improved the convenience of the on‐site detection of food contaminants during food safety evaluation. A similar target‐responsive DNA hydrogel was integrated with a pH meter for the readout to develop a portable sensor for Aflatoxin B1 (AFB1) detection.^[^ ^195^ ^]^ The AFB1‐responsive aptamer‐cross‐linked hydrogel collapsed due to the target AFB1 binding to its specific aptamer with high affinity, resulting in the release of trapped urease into the solution. This process triggered an enzyme hydrolyze reaction of urea and elevated the pH value due to the generation of carbon dioxide and ammonia (Figure 7D). The proposed portable device was applied to detect the pH value while building a direct relationship with the AFB1 concentration (Figure 7E). In the assessment of selectivity, no matter coexisting with other interferent toxins or in the real food samples, a distinct change in pH value only appeared when in the presence of target AFB1, which revealed this strategy can effectively avoid other interferents of the complex matrix to the effects on the detection of AFB1. Contrarily, Hao et al.^[^ ^196^ ^]^ investigated a novel sensitive fluorescent DNA hydrogel aptasensor incorporated with RCA technology for OTA detection in beer. Relying on a similar competitive binding mode of three essential elements consisting of the aptamer, complementary sequence, and target, the DNA hydrogel that was self‐assembled with the support of RCA was applied for the rapid, simple, and sensitive detection of OTA. Even though the same types of food contaminants are involved, the DNA hydrogel can be combined with different sensing elements or other biotechnologies to build various types of sensors. Accordingly, DNA hydrogels provide outstanding flexibility and diversity to the biosensing field.

#### 4.1.2 Treatment and Analysis of Environmental Pollutants

**Figure 8:**
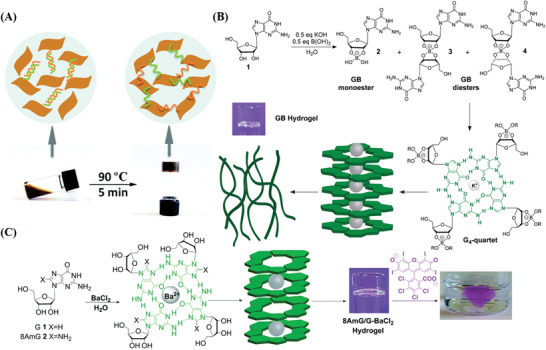
Examples of smart and functionalized NAHs for micropolluted water treatment. A) The procedure for preparing GO/DNA SH and the proposed gelation mechanism. Reproduced with permission.^[^ ^76^ ^]^ Copyright 2010, American Chemical Society. B) The proposed mechanism for the gelation of water by G 1 and KB(OH)_4_, via the formation of GB borate diesters 3/4, followed by the formation and stacking of G4·M^+^ quartets and the intermolecular association with G4‐wires. Reproduced with permission.^[^ ^201^ ^]^ Copyright 2015, American Chemical Society. C) The binary 1:1 mixtures of G 1 and 8AmG 2 with alkali/alkaline earth salts (K^+^ and Ba^2+^), resulting in G4‐quartet structures that lead to the formation of stiff, stable, and transparent hydrogels. The 8AmG/G‐BaCl_2_ hydrogel can selectively extract anionic dyes from solutions in the gel phase. Reproduced with permission.^[^ ^202^ ^]^ Copyright 2017, The Royal Society of Chemistry.

NAHs are environmentally favored due to their numerous excellent properties, such as large surface areas, porous structures, rich functional groups, high adsorption ability, multiple optional FNAs, programmability, ecofriendliness, and security. In the currently developed hydrogel systems, such biomaterials have been identified as adsorbents,^[^ ^197^ ^]^ immobilization carriers,^[^ ^198^, ^199^ ^]^ and catalysators,^[^ ^39^, ^41^, ^200^ ^]^ providing advanced technology for wastewater disposal and displaying high specificity for the analysis of environmental pollutants. In fact, DNA hydrogels are particularly useful for the treatment of micropolluted water, which refers to various kinds of water with low‐content pollutants or pollution factors, such as that found in mutagenesis, carcinogenesis, and teratogenesis.^[^ ^54^ ^]^ Compared to carbon‐based materials, clay minerals, metallic materials, and other common materials for wastewater treatment, DNA hydrogels adequately meet the requirements for the treatment of micropolluted water in terms of easy degradation and low toxicity, which had emerged in many early studies. Most of them focused on pollutant separation mainly through hybrid DNA hydrogels consisting of synthetic polymers or nanomaterials.^[^ ^54^ ^]^ For example, a smart GO/DNA composite, a self‐assembled hydrogel with high mechanical strength, excellent environmental stability, high dye‐adsorption capacity, and self‐healing functions has proven effective for dye adsorption at a level that is even superior to that of carbon nanomaterials to some extent^[^ ^76^ ^]^ (**Figure** **8**A). In addition, smart FNA‐based hydrogels with 3D network conformation can respond to environmental factors or specific biomolecules and trigger the volume‐change or state‐transition. The application of G4‐based hydrogels is a good example. Generally, supramolecular G4‐based hydrogels are fabricated using a combination of guanosine (G 1) or its derivative, 8‐aminoguanosine (8AmG 2), with stoichiometric concentrations of cations, which play a crucial role in stabilizing the conformation of the G4‐quartet ligands, such as K^+^, M^+^, and Ba^2+[^ ^76^, ^201^ ^]^ (Figure 8B). The G4 structure provides these transparent hydrogels with the ability to selectively bind cationic or anionic dyes by adjusting the salt levels involved in the formation of the gels, many of which are wastewater pollutants^[^ ^202^ ^]^ (Figure 8C). Moreover, the strong permeability and porous structure of DNA hydrogels can effectively encapsulate and immobilize pollutants, largely solving the problem of sludge clogging and sewage reclamation during wastewater treatment. Therefore, DNA hydrogels are effectively applied during environmental analysis, while realizing either the direct or indirect treatment and assessment of low‐level pollutants, especially heavy metals, and persistent organic pollutants.

#### 4.1.3 Bioimaging

**Figure 9:**
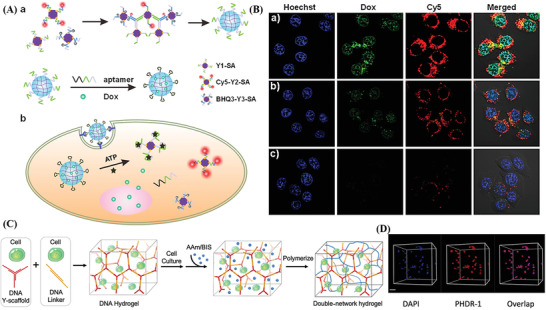
Smart and functionalized NAHs‐supported bioimaging applications. A) Illustration of ATP‐triggered Dox‐SDH‐Apt imaging and targeted‐therapy system. B) CLSM images of different‐pretreated MCF‐7 cells with Dox‐SDH‐Apt system. Reproduced with permission.^[^ ^204^ ^]^ Copyright 2019, American Chemical Society. C,D) Construction and 3D cell imaging of DNA‐PAM/BIS dual‐network hydrogel. Reproduced with permission.^[^ ^206^ ^]^ Copyright 2020, American Chemical Society.

Bioimaging technology is the most direct and effective method for biological structure and function research. The development of information technology and the application of new nanomaterials, such as NAHs, cause bioimaging technology to play a significant role. Even though they are in the initial stage of development, the versatile and responsive NAHs present significant potential in the field of bioimaging. Currently, hybrid DNA hydrogels are primarily developed for fluorescence imaging by combining nanomaterials or biomolecules with unique functions. This provides a method for obtaining information by collecting fluorescent signals after introducing fluorescent biological materials into the body. For example, a smart and versatile Au‐DNA hydrogel (AuDH) system for the simultaneous and sensitive imaging of intracellular multiplex miRNAs was constructed using a toe‐hold strand‐displacement reaction and hairpin‐locked, DNAzyme‐assisted, miRNA recycling dual‐signal amplification with loading active metal ions (AuDH/M^n+^/H).^[^ ^203^ ^]^ This hydrogel system successfully and sensitively monitored the changes regulated by siRNA or miRNA mimics in living cells. Furthermore, this strategy enables the accurate and sensitive differentiation of cancer cells by imaging intracellular multiplex miRNAs even at exceedingly low expression levels, which proves useful for the diagnosis and prognosis of cancer.^[^ ^203^ ^]^ Therefore, to be useful imaging probes or drug carriers, the typically bulky size of DNA hydrogels has been addressed using an innovative, yet simple, design approach that developed a new DNA nanohydrogel for activable imaging and therapy in targeted cancer cells.^[^ ^204^ ^]^ The streptavidin (SA)‐scaffolded DNA nanohydrogels (SDH) constructed via a direct DNA self‐assembly using three types of SA‐based DNA tetrad made it possible to finely control their size within a nanoscale range, which provides favorable carriers for intracellular imaging and transport (**Figure** **9**A). By further incorporating therapeutic agents, doxorubicin (Dox) and the tumor‐targeting MUC1 aptamer (Apt), these multifunctionalized Dox‐SDH‐Apt systems can specifically target cancer cells and selectively release the preloaded therapeutic agents via structure switching when in an ATP‐rich intracellular environment, leading to the activation of the fluorescence and efficient treatment of cancer cells^[^ ^204^ ^]^ (Figure 9B). Moreover, DNA supramolecular hydrogels have also attracted significant attention as substitutes for native extracellular matrices (ECM) due to their excellent permeability, modularity, responsiveness, and tunable mechanical properties, providing cells with essential 3D support.^[^ ^19^, ^198^, ^205^ ^]^ Generally, the observation of cell morphology and functions in situ involves immobilization, labeling, and imaging processes requiring excellent stability from the hydrogels during washing and immersion. Consequently, to improve the stability of the hydrogels for better imaging, a covalent secondary network was built in the DNA supramolecular hydrogel via in situ polymerization with a PAM/*N,N*‐bisacrylamide (BIS) network, while successfully constructing a stable 3D transparent system for cell culture and observation^[^ ^206^ ^]^ (Figure 9C). Due to the efficient enhancement of the mechanical properties and the immobilization of the cells inside the hydrogel, this strategy is suitable for immunostaining and cell imaging (Figure 9D). Furthermore, it is expected that multimodal fusion combined with NAHs will represent the development trend of bioimaging systems.

### 4.2 NAHs for Biomedicine

#### 4.2.1 Controllable Drug Delivery and Targeted Therapy

NAHs have been brought into sharp focus in the fields of drug delivery and targeted therapy. Notably, their inherent unique conformation and excellent biological characteristics have been concretely reflected in drug loading, delivery, and release.^[^ ^207^ ^]^ 1) The highly porous structure and swelling properties of NAHs which directly influence the drug loading effects are easily adjustable by controlling the density of cross‐links in the hydrogel matrix or changing the affinity of the hydrogels to the aqueous environment. Furthermore, the porosity of the hydrogel network also permits drugs loading into the gel matrix or conjugating to the hydrogel scaffolds. Subsequent drug release at a rate will be dependent on the degradation rate of nucleases and the diffusion coefficient of the small molecules or macromolecules throughout the gel network.^[^ ^207^, ^208^ ^]^ 2) Biocompatibility is promoted by the high‐water content of the hydrogels and the physiochemical similarity of hydrogels to the native ECM.^[^ ^209^ ^]^ 3) The biodegradability or dissolution that forms part of the hydrogel design is generally achieved by responding to specific stimuli, such as enzymatic, hydrolytic, or environmental factors.^[^ ^207^ ^]^ 4) Nucleic acids with unique programmability, diverse chemical modification, as well as specific spatial structures and functions, make it an ideal carrier for drug delivery. At present, NAHs as a promising controllable delivery system has been applied to load several types of cargoes, such as nucleic acid drugs, small‐molecule drugs, and other specific biomaterials or tools, which mainly perform their functions in gene therapy,^[^ ^210^, ^211^, ^212^, ^213^ ^]^ immunotherapy,^[^ ^43^, ^214^, ^215^, ^216^, ^217^ ^]^ and chemotherapy.^[^ ^86^, ^208^, ^218^, ^219^, ^220^, ^221^ ^]^

**Figure 10:**
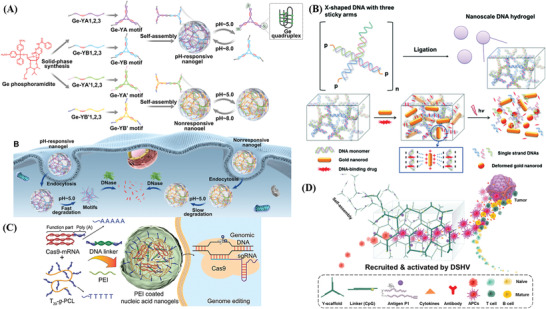
Drug delivery and targeted therapy platforms based on multiple smart and functionalized DNA hydrogels. A) pH‐responsive Ge‐contained NAHs for efficient chemodrugs delivery. Reproduced with permission.^[^ ^219^ ^]^ Copyright 2019, American Chemical Society. B) AuNR‐embedded DNA hydrogel nanoscaffold loaded with Dox for chemotherapy. Reproduced with permission.^[^ ^220^ ^]^ Copyright 2015, The Royal Society of Chemistry. C) Cas 9‐mRNA embedded NAHs for genome editing. Reproduced with permission.^[^ ^212^ ^]^ Copyright 2020, American Chemical Society. D) NAH‐based immune therapeutical vaccine system. Reproduced with permission.^[^ ^214^ ^]^ Copyright 2018, American Chemical Society.

In recent years, with deep investigations of the microenvironment alteration near different lesions, the treatment protocols with smart administration systems have made great progress in the delivery of nucleic acid drugs, chemodrugs, and certain small drugs. The stimuli‐responsive DNA hydrogels as novel smart delivery carriers have come to the foreground and several environment triggers have been explored for driving the drug delivery systems, such as pH,^[^ ^219^ ^]^ light,^[^ ^220^ ^]^ and magnetic field.^[^ ^84^, ^86^ ^]^ In the newly‐reported study of Zhang et al.,^[^ ^219^ ^]^ they constructed a pH‐responsive NAH with gemcitabine (Ge)‐i‐motif which was synthesized by replacing deoxycytidine (dC) with its nucleoside analog Ge (**Figure** **10**A). Ge as an effective anticancer drug and a key functional unit in the assembly and disassembly of NAHs played a dual role in this study. On the one hand, Ge has been used to the clinic treat cancers of the pancreas, lung, ovary, and breast. On the other hand, due to the similar structure between Ge and dC, the Ge‐i‐motif also can fold to the quadruplex structure under the acidic condition. Thus, the structure of Ge‐containing NAHs was switchable by responding to pH changes of intracellular microenvironment after cellular uptake, thereby causing the rapid enzymatic degradation of NAHs with high‐efficient drug release to surge anticancer activity inside cells. In addition, Song et al.^[^ ^221^ ^]^ constructed a light‐triggered DNA hydrogel as a scaffold to assemble gold nanorods (AuNRs) with Dox in nanoscale proximity by using electrostatic and DNA binding interactions to overcome the limited conjugation capability of AuNRs (Figure 10B). The experiments on cellular and animal models have demonstrated a highly synergistic combined cancer therapy. Compared to direct loading, the introduction of FNAs and the RCA technique makes it possible to target cancer cells, while providing satisfactory biostability and a simple fabrication process to realize stimuli‐responsive drug delivery in a tumor microenvironment. Yao and Yuan^[^ ^86^ ^]^ constructed a magnetic DNA nanogel for applications targeting drug delivery and triggering drug release. Focusing on magnetic nanoparticles, a DNA nanogel layer was synthesized using the RCA technique whose products equip the various sites for the loading of anticancer drugs. Driven by an external magnetic field, the magnetic DNA nanogel can target tumor cells efficiently. The DNA nanogel displayed a proven multistimuli‐responsiveness and primarily included temperature, pH, and nuclease, enabling a controlled release of anticancer drugs. This system achieves magnet‐controlled drug delivery and stimuli‐triggered drug release while providing a new strategy for the development of precision medicine. Inspired by the fact that soft organisms can easily access confined spaces, another super‐soft, super‐elastic magnetic DNA hydrogel‐based robot was designed and constructed by the same team.^[^ ^84^ ^]^ This DNA robot was stabilized and consisted of a combinational dynamic and permanent cross‐linking network through chain entanglement and DNA hybridization, which resulted in shear‐thinning and cyclic strain properties. Consequently, it presented shape‐adaptive properties, enabling magnetically driven navigational locomotion in confined and unstructured spaces by rapidly deforming and recovering its shape, allowing the DNA robot to work as a smart vehicle for the delivery of living cells. The interaction between the drug molecules and the DNA provided the DNA robot with the ability to absorb a chemotherapy drug under static, as well as flow conditions. The DNA robot shows promise for contributing to in vivo applications such as diagnosis‐therapy, implantable medical devices, and minimally invasive surgery, and even undertaking more complex tasks in biomedical‐related fields. Most recently, another type of smart hybrid DNA hydrogel was created for the delivery of therapeutic molecules to restore the functionality of damaged tissues.^[^ ^220^ ^]^ The study designed a nanocomposite DNA‐based hydrogel cross‐linked with oxidized alginate (OA) through the formation of reversible imine linkages. The formulated hydrogel with optimized ratios functioned as an injectable carrier for the sustained delivery of a small molecule drug, simvastatin, for more than a week. After confirming the bioactivity of the released drug, it was revealed that DNA could be used as a natural biopolymer to fabricate self‐healing injectable hydrogels with sustained‐release properties for minimally invasive therapeutic approaches.

NAHs because of outstanding biocompatibility, structural stability, and easy construction process have become a favorable novel delivery vector for transporting nucleic acid drugs or tools into the target cells to regulate gene expression and complete gene therapy. In recent years, the research team of Zhang et al. successively reported several different nucleic acid nanogel‐based delivery systems for gene regulation and genome editing.^[^ ^210^, ^211^, ^212^, ^213^ ^]^ Xue et al.^[^ ^210^ ^]^ directly applied DNA tetrahedron as building blocks and small interfering RNA (siRNA) as cross‐links to fabricate all‐nucleic acid nanogels for the target gene knockdown. DNA tetrahedron with advanced self‐assembled nanostructure protected siRNA from biodegradation by nuclease. In the meanwhile, the fabrication process neither the usage of toxic agents nor the introduction of other extraneous synthetic materials avoided the toxic effects to cells and ensured biocompatibility of intracellular delivery.

Except for the representative therapeutic agent siRNA, the booming development of CRISPR (clustered regularly interspaced short palindromic repeats)/Cas (CRISPR‐associated) system has generated considerable attraction in the diagnosis and therapy of diseases. The research team of Zhang et al. developed two NAH‐based delivery systems by different strategies.^[^ ^211^, ^212^ ^]^ One was to codelivery of Cas 9 and single‐guide RNA (sgRNA) complex which was loaded into a first‐synthetized DNA‐grafted polycaprolactone brush (DNA‐g‐PCL) by Ding et al.^[^ ^211^ ^]^ in 2019. The Cas 9/sgRNA complex was gradually encapsulated with continuous cross‐linking mediated by DNA links into a noncationic NAH which was different from common‐used cationic carriers which may cause toxic accumulation from transfect agents and easy digestion by nuclease. The Cas 9/sgRNA‐embedded NAHs to the model Hela cell efficiently completed the cleavage of the EGFP coadding region, thereby knocking down the target gene expression. The other strategy was to deliver Cas 9 protein by integrating its code information into message RNA (mRNA) which plays an important role in regulating therapeutic protein expression in the target cell for the diseases treatment and provides solid assurance of stability for the delivery system in the absence of nuclease. Huang et al.^[^ ^212^ ^]^ followed this strategy and achieved the first delivery of the functional mRNA by NAHs. According to a similar route as the previous study, the polythymine (poly T)‐g‐PCL as a DNA brush synthesized first and hybridized with the polyadenine (poly A) tail of the Cas 9‐encoded mRNA. Subsequently, DNA linkers were incorporated to cross‐link and form the mRNA‐NAHs (Figure 10C). However, compared with the non‐cationic NAHs, a cationic polyethylenimine (PEI) was coated to the surface of mRNA‐NAHs for facilitating the endocytosis and endosomal escape. The optimized Cas 9‐mRNA NAHs were capable to induce genome editing with high efficiency and meanwhile, the evaluation results in their cytotoxicity suggested the PEI‐coated NAHs delivery system also possessed high biocompatibility. Therefore, the progressive NAH‐based delivery platforms in gene therapy are appropriate vectors to transport therapeutic nucleic acids and functional proteins for genome editing and protein expression regulation.

As the most powerful system for preventing cancerous cells from propagating, the reactivation of the immune system is effective in killing cancer cells, which also leads to the extensive use of immunostimulatory agents in cancer chemo/immunotherapy.^[^ ^222^ ^]^ Unmethylated cytosine‐phosphate‐guanine (CpG) dinucleotide, or the CpG motif, is a well‐known pathogen‐associated molecular pattern that denotes a danger signal for the invasion of pathogens, like bacteria.^[^ ^223^ ^]^ Therefore, the delivery of immunostimulatory signals and anticancer agents is crucial for the stimulation of both innate and adaptive immune responses.^[^ ^217^ ^]^ DNA is a promising polymer that can be used as a delivery system, a combination that would be highly effective for tumor chemo/immunotherapy.^[^ ^217^ ^]^ As such, a series of CpG DNA hydrogels has been designed and fabricated. Nishikawa et al.^[^ ^217^ ^]^ designed a new, highly immunostimulatory DNA unit by incorporating six highly potent CpG motifs into X‐DNA, and prepared CpG DNA hydrogels by ligation using a DNA ligase. Dox was intercalated into the DNA to form Dox/CpG DNA hydrogels for the sustained delivery of a variety of anticancer agents and antigens for cancer immunotherapy. Based on previous studies, the same team continued to achieve sustained OVA release from a DNA hydrogel via the cationization of the antigen.^[^ ^215^ ^]^ Ethylenediamine (ED)‐conjugated cationized OVA (ED‐OVA) was used to slow down the speed of the release. The results demonstrated that ED‐OVA mixed with CpG DNA hydrogels efficiently binds to mouse dendritic DC2.4, resulting in high antigen presentation. Intratumoral injections of the ED‐OVA/CpG DNA hydrogel significantly delay the tumor growth of OVA‐expressing EG7‐OVA cells in mice. Consequently, the animal experimental results indicated that a vaccine consisting of immunostimulatory CpG DNA hydrogel and cationized antigens could be effective for cancer immunotherapy. This team further developed a Takumi‐shaped DNA hydrogel (iTakumiGel) consisting of two partially complementary oligodeoxynucleotides (ODNs) to deliver the immunoinhibitory ODNs (INH‐ODNs), which are potent inhibitors of Toll‐like receptor 9 (TLR9) activation.^[^ ^216^ ^]^ The iTakumiGel effectively increased the cellular uptake of INH‐ODN and proved useful for the delivery of INH‐ODNs to immune cells to inhibit TLR9‐mediated hyperinduction of proinflammatory cytokines. Besides, Shao et al.^[^ ^214^ ^]^ assembled Y‐scaffold, CpG‐immobilized linker, and antigen P1 to construct an injectable NAH‐based tumor vaccine system of which the working mechanism in vivo imitated a lymph node that the raised local concentration of CpG activated and recruited antigen‐presenting cells (APC) to enhance immune response and anticancer effects. This NAH‐base therapeutical vaccine system can be developed into a general platform for benefit of multiple diseases therapy (Figure 10D).

**Figure 11:**
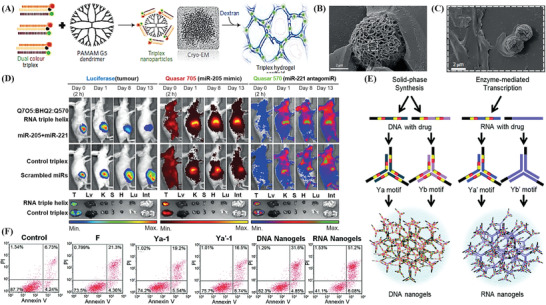
Performance of smart and functionalized RNA hydrogels in controllable drug delivery and targeted therapy. A) Self‐assembly route and B,C) SEM characterization images of RNA‐triplex hydrogel scaffold. D) In vivo imaging of model mice for monitoring the targeted tumor therapeutic efficacy of RNA‐triplex hydrogel scaffold. Reproduced with permission.^[^ ^224^ ^]^ Copyright 2019, Springer Nature. E) Schematic illustration of self‐assembly ways of F‐containing DNA nanogels and RNA nanogels, and F) cancer cells apoptosis behaviors induced by them. Reproduced with permission.^[^ ^225^ ^]^ Copyright 2018, The Royal Society of Chemistry.

Even though the studies of RNA‐based hydrogels were at the primary stage, their promise cannot be neglected. To construct efficient delivery vehicles for the therapeutic applications of miRNA (miR) in cancer, the RNA building blocks are self‐assembled into a dual‐colored RNA‐triple‐helix structure comprising two miRNAs, referring to the miR mimic (tumor suppressor miRNA) and an antagomiR (oncomiR inhibitor).^[^ ^224^ ^]^ Both provide the outstanding capability to abrogate tumors synergistically. The stable triplex nanoparticles formed via conjugation of RNA triple helices to polyamidoamine (PAMAM) G5 dendrimers were further assembled to form an RNA‐triple‐helix adhesive scaffold upon interaction with dextran aldehyde (**Figure** **11**A,C). These assembled products could chemically interact and adhere to natural tissue amines in the tumor. The results of a triple‐negative breast cancer mouse model indicated that conjugates retained their functionality, both in vitro and in vivo, exhibiting efficient anticancer effects (Figure 11D). This study provided a foundation for subsequent research on RNA‐based hydrogels. In addition, based on the structural similarity between the nucleoside analog, floxuridine (F), and the natural nucleoside, thymidine (T), F can be incorporated into nucleic acid strands via either solid‐phase synthesis or enzyme‐mediated transcription^[^ ^225^ ^]^ (Figure 11E). In the meanwhile, F is a promising cytotoxic nucleoside analog for precisely‐targeted cancer therapeutics. Thus, the synthesized F‐integrated DNA or RNA strands can not only retain molecular recognition but can also be used as building units to be further assembled into nucleic acid‐based spherical nanogels, which were efficiently taken up by cells, after which the therapeutic agents were released in the presence of nuclease. The results revealed that the F‐containing nucleic acid nanogels exhibited excellent ability to induce cell apoptosis to further inhibitory activity against cancer cells (Figure 11F). These composite DNA–RNA nanogels are an innovative way of integrating the superiority of nucleic acids while satisfying the strict requirements of in vivo applications.

#### 4.2.2 Cell Cultivation and Tissue Healing

The simple preparation of the DNA hydrogel and its wide scope of applications provide new perspectives for various cell study challenges and tissue engineering. The attachment and proper functioning of the cells in most mammalian tissues rely on an ECM. In vitro cell cultures, an artificial ECM with the most appropriate properties, need to be chosen for each application. Early studies mostly adopted gelatin, polyethylene glycol (PEG), or other gelling materials as the main backbone and further incorporated aptamers via free radical polymerization to produce a variety of functionalized hydrogels with the properties of targeting and molecular recognition. In the study of Chen et al.,^[^ ^226^ ^]^ an aptamer‐functionalized PEG hydrogel can achieve effectively cell type‐specific adhesion which was affected by the aptamer concentration, the spacer length, and the cell seeding time. In contrast, the addition of complementary oligonucleotides can attenuate cell adhesion by blocking the binding between aptamers and receptors.^[^ ^226^ ^]^ Thus, the aptamer‐PEG hydrogels as a promising biomaterial may mimic the functionals of ECM provide cells with essential growth factors, which was further evident in the study of Zhang et al.^[^ ^227^ ^]^ They integrated gelatin, PEG, and aptamers to synthesize a hybrid hydrogel possessing with macroporous structure and high permeability for molecular transport. The results of model experiments showed that the aptamer‐gelatin hydrogel can sustainably release the loaded grow factors and effectively maintain higher bioactivity during 14 days, which provided necessary conditions for the culture of cells loaded into the hydrogel.^[^ ^227^ ^]^ Thus, the aptamer‐chimeric hydrogel as the initial form of NAHs has been developed as a promising biomaterial in cell adhesion and cultivation.

**Figure 12:**
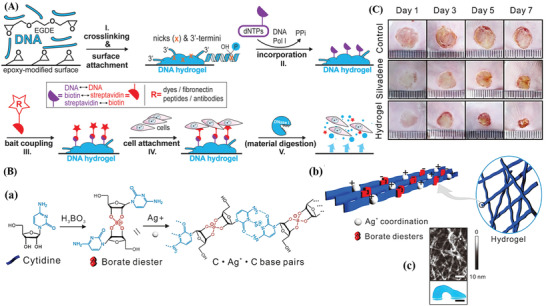
Examples of smart and functionalized NAHs applied for cell cultivation and tissue healing. A) Preparation routes and working principle of bait‐decorated DNA hydrogels. Reproduced with permission.^[^ ^228^ ^]^ Copyright 2019, Wiley‐VCH. B) Formation of a C‐B‐C·Ag^+^‐based metallonucleoside hydrogel and its induction of wound healing to mice models. Reproduced with permission.^[^ ^229^ ^]^ Copyright 2019, American Chemical Society.

For exploring more possibilities in the design of structures, DNA hydrogel is also a viable candidate for being developed as a promising ECM mimic due to its hydrophilic character, programmability according to the sequence design, and susceptibility to enzyme modification. Therefore, this denotes a chemically cross‐linked DNA hydrogel that can be readily enzymatically modified to incorporate functional moieties that allow the material to be recognized explicitly by the desired cells.^[^ ^228^ ^]^ Compared with synthetic oligonucleotides, the less expensive bulk salmon sperm DNA was selected to prepare bait‐decorated DNA hydrogels which were functionalized via a DNA polymerase‐catalyzed nick translation reaction to incorporate modified nucleotides, allowing for the installation of easily identifiable molecules that mediate cell adhesion. In the presence of specific DNA‐cleaving enzymes, the cell detachment was investigated by degrading the hydrogel under mild conditions (**Figure** **12**A). This approach opens the door for applications where cells can be specifically captured and released from DNA hydrogel‐coated surfaces or when cells need to be detached without proteolytic damage.

Except for direct cell cultures, injectable and biocompatible DNA hydrogels are also being increasingly utilized as scaffolds for in situ tissue engineering and wound healing. The 3D DNA hydrogel architecture for the regeneration of injured or diseased tissues meet the requirements for promoting cell growth, providing mechanical support, and serving as a conduit for nutrients during new tissue growth. Tang et al.^[^ ^229^ ^]^ created a functional supramolecular DNA hydrogel by mixing cytidine (C) with a half equivalent each of B(OH)_3_ and AgNO_3_. Based on the orthogonal formation of the C·Ag^+^·C dimer, this supramolecular could rapidly switch between the sol and gel states and was used to promote the closure of burn wounds in a mouse model (Figure 12B). Compared with the novel bioadhesive/gel systems that can prevent the skin penetration behavior of UV filters,^[^ ^230^ ^]^ this soft, wet, and biocompatible C‐B‐C·Ag^+^ supramolecular hydrogel can provide further healing and protection, making it an ideal material for the promotion of wound closure (Figure 12C). Given the unique combination of ease and low cost of preparation, long‐term stability, injectability, antibacterial activity, and wound‐closure propensity, this hydrogel is likely to be suitable for other applications in regenerative medicine and tissue engineering.

#### 4.2.3 Biomimetic Materials and 3D Bioprinting

**Figure 13:**
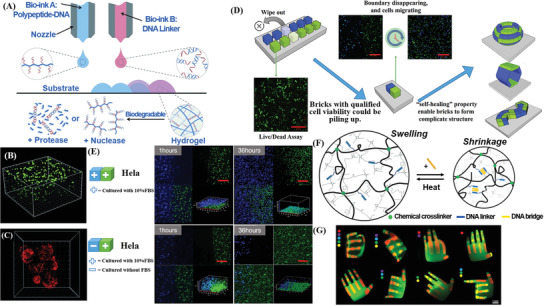
Examples of smart and functionalized NAHs applied for biomimetic materials and 3D bioprinting. A) Fabrication of a polypeptide–DNA hydrogel and B,C) that containing AtT‐20 cells by 3D bioprinting. Reproduced with permission.^[^ ^15^ ^]^ Copyright 2015, Wiley‐VCH. D) Scheme of the brick‐to‐wall technology based on DNA hydrogels for tissue structure simulation. E) Evaluation of cell migration ability in DNA‐gel bricks. Reproduced with permission.^[^ ^234^ ^]^ Copyright 2017, American Chemical Society. F) Illustration of reversible cross‐linking of a D‐gel. G) Fluorescent images of D‐gel palm with DNA‐triggered movements. Reproduced with permission.^[^ ^235^ ^]^ Copyright 2019, Wiley‐VCH.

Bioprinting is an emerging technology with various applications for producing functional tissue constructs to replace injured or diseased tissues. It is a relatively new approach that provides high reproducibility and precise control over the fabricated tissue‐like structures in an automated manner, potentially enabling high‐throughput production.^[^ ^231^ ^]^ During the bioprinting process, a solution of a biomaterial or a mixture of several biomaterials in a hydrogel form, usually encapsulating the desired cell types, termed the bioink, is used for creating tissue constructs. This bio‐ink can be cross‐linked or stabilized during or immediately after bioprinting to generate the final shape, structure, and architecture of the designed construct.^[^ ^232^ ^]^ Bioinks can be manufactured from natural or synthetic biomaterials alone or a combination of the two as hybrid materials. In some instances, cell aggregates without any additional biomaterials can also be used as a bio‐ink in bioprinting processes. An ideal bioink should possess adequate mechanical, rheological, and biological properties of the target tissues, which are essential to ensure the correct functionality of the bioprinted tissues and organs.^[^ ^233^ ^]^ In fact, non‐covalently cross‐linked hydrogels have been widely explored as scaffold materials due to their similarities to natural ECM, thus, providing structural and physical support for cells like those found in the natural environment. Notably, NAHs provide an excellent building scaffold for the construction of versatile devices and materials possessing specific biodegradability and biocompatibility features, as well as self‐healing interlayer abilities, and has been applied for in situ multilayer 3D bioprinting with living cells. In 2015, Li et al.^[^ ^15^ ^]^ first reported a method for rapid in situ multilayer 3D bioprinting with a supramolecular polypeptide–DNA hydrogel as the bio‐ink (**Figure** **13**A). The excellent healing properties of printed hydrogels benefited from the dynamic cross‐linking by DNA hybridization, which resulted in geometrically uniform constructs without boundaries. Furthermore, the printed structures can retain their shapes up to a millimeter scale without collapsing, benefiting from the high mechanical strength and the non‐swelling/shrinking properties of the hydrogel. This strategy has been applied for cell printing and has been proven to produce structures containing viable cells with normal cellular functions (Figure 13B,C). Considering that the hydrogel is biocompatible with cells, permeable to nutrients, and biodegradable, the same team further studied the printable biomaterial for the fabrication of complex 3D tissue‐like constructs during tissue engineering. Wang et al.^[^ ^234^ ^]^ illustrated a new “brick‐to‐wall” technology based on the unique properties of DNA supramolecular hydrogels to fabricate 3D tissue‐like structures by encapsulating different cell types in DNA hydrogel bricks, which are then combined to build 3D structures (Figure 13D). The signal responsiveness of the cells via the DNA gels was evaluated and revealed that the gel permitted cell migration in three dimensions (Figure 13E). Therefore, this convenient, effective, and reliable strategy for cell manipulation is crucial for the further development of artificial tissue‐like structures. In the most recent research, a novel strategy was proposed that magnified the nanometer scale DNA self‐assembly into macroscopic mechanical responsiveness, while 3D printing technology played an important role in this process. By incorporating molecularly engineered linker DNA sequences into a chemically cross‐linked polymeric network, a mechanically responsive material was created, which was denoted as a double cross‐linking DNA hydrogel (D‐gel)^[^ ^235^ ^]^ (Figure 13F). Therefore, to take a step closer to manufacturing, this hydrogel was combined with a bottom‐up 3D printing technology to achieve programmable reconfiguration and directional movement in macroscopic objects, which can even mimic the complex gestures of human hands, which is potentially useful in soft robotics (Figure 13G).

## 5 Conclusions and Perspectives

With the synchronous advances of DNA nanotechnology, nucleic acids contribute unique genetic information and brilliant attributes as a powerful support for creative works in biomaterial science. NAHs, as a rising star, are aimed at smart and functional development. Stimulus‐induced DNA hydrogels undergoing volume, color, and phase changes when triggered by specific factors have been investigated for the design of biosensors, drug delivery systems, environmental analytical media, cellular scaffolds, and other roles in bioanalytical and biomedical applications. Furthermore, the incorporation of novel nanomaterials has dramatically enriched the functions and improved the properties of NAHs. Notably, the association of mature technologies or materials with NAHs has made significant progress in broadening their application fields, such as surface‐enhanced Raman scattering technology,^[^ ^236^ ^]^ low field nuclear magnetic sensing technology,^[^ ^237^ ^]^ and fluorescent MoS_2_ quantum dots.^[^ ^238^ ^]^ Although these studies are still in the methodology establishment stage, they represent promising potential uses of NAHs.

Despite the tremendous progress in developing NAHs, to further drive the applications forward, several challenges involving preparation improvement, function promotion, and commercial application development must be addressed.

i) Improve NAHs assembly efficiency: To promote the sustainable development of smart NAHs in manufacturing, the key is to keep adjusting and updating the advanced, economical, and highly efficient preparation and construction methods in light of the realistic development needs. On the one hand, exploring additional methods for the modification of nucleic acids, as well as introducing other techniques to supplement the current nucleic acid amplification technologies will benefit hydrogels regarding the enrichment of assembly modes, the increase of drug delivery forms, and the adjustability of the degradation periods in vivo. For example, catalytic hairpin assembly (CHA) is another enzyme‐free nucleic acid‐activated chain reaction of HCR. CHA mediates the assembly of the well‐designed nucleic acid hairpin structures activated by triggering an initiator strand to generate stable and advanced dsDNA nanoscale assemblies.^[^ ^238^, ^239^ ^]^ It has been developed for amplified sensing and programmed nanostructuring and may present another promising method for the preparation of NAHs. Also, the introduction of RNA or ribonucleotides will undermine the dominance of DNA in NAHs. Combined with rolling circle transcription (RCT) techniques, RNA interference (RNAi) technologies, as well as folded motifs with a precise 3D shape based on canonical Watson–Crick and non‐canonical base pairs, RNA hydrogels are expected to propel NAH materials to new heights.^[^ ^240^ ^]^ On the other hand, the screening and tailoring of more abundant FNA‐based building blocks, different research objects, or targets will essentially promote the richness, intelligence, and application feasibility of NAHs in assembly and design. In the meantime, it is worth examining that the specific FNA sequences of different research objects or targets may unexpectedly affect the assembly and application processes, such as the promotion or inhibition of a reaction, the changes in mechanical strength, and the weakening or enhancement of the visible properties.
ii) Achieve NAHs precise disassembly: To better serve the applications in vivo, the disassembly manners of NAHs are necessary to be investigated. Building on a comprehensive understanding of cross‐linking mechanisms, stimuli‐responsive mechanisms, and kinetics, the assembly, and disassembly of the functional building blocks of the NAHs will be controllable in terms of specific structures or shapes via flexible operations on either a microscopic or macroscopic scale. Furthermore, the size, surface charge, and hydrophobicity, as well as other physicochemical properties of these functional moieties can also be customized through proper fine‐tuning to meet the specific demands, such as assembling efficiency, improving target ability, and drug‐releasing rate. The ability to be freely and widely adjustable will allow expansion to more application fields to satisfy different needs, especially in vivo.
iii) Develop customizable smart NAHs: The essential elements of practical smart NAHs include a variety of building blocks, properties, and stimuli collected from biomaterials and environmental conditions which can be regarded as different types of components with respective functions in a toolbox. In the future, the modularization design and pipeline technology may be adopted with selecting appropriate components from this content‐rich toolbox to achieve customizable production to develop novel smart NAHs. This is closely associated with the innovation of technologies, the incorporation of nanomaterials, as well as the investigation of various stimuli and environmental factors. Nowadays, the integration of nanomaterials, such as metal or semiconductor nanoparticles or nanorods, or carbon materials with stimuli‐responsive hydrogel can yield hybrid matrices for controllable stiffness properties, such as interfaces for the dictated growth of cells. In future studies, nucleic acids, especially FNAs, can be further combined with bioactive components or small molecules via chemical modification, as well as novel nanomaterials via encapsulation or embedment. This can directly decrease the consumption and cost of nucleic acids, improve the plasticity and stability of the hydrogel matrix, and enhance the functionality and stimuli responsiveness of hydrogels. For example, Tan et al. have synthesized several different uniform spherical nucleic acids by conjugating specific aptamers with lipid micelles (DLMs) or prodrugs. The former strategy for the synthesis of nanoassembly based on the covalent linking of aptamer and lipid units via a photoinduced polymerization reaction, enhanced the entire stability of the DNA–lipid micellar structures, further improving the targeting ability of the aptamer.^[^ ^241^ ^]^ The latter, used for the construction of aptamer‐prodrug conjugate (ApPdC) micelle, generated a synergistic chemodynamic therapy (CDT) effect via cascading bioorthogonal reactions without relying on either the endogenous H_2_O_2_ content or strongly acidic conditions.^[^ ^242^ ^]^ These advanced FNA structures still represent a void in the applications of NAHs. Moreover, the assembling methods will convert from basic and simple strategies to layer‐by‐layer and sophisticated strategies. The application modes will no longer be primarily in vitro but will be more focused on eliminating the inherent limitations of in vivo applications, including, but not limited to, cellular intake, toxicity elimination, bioactivity protection, as well as oral and local drug delivery methods beyond injection.
iv) Build interdisciplinary cooperation by technology integration for NAHs development: These days, the progress of interdisciplinary research is followed with interest, while the enhanced communication and cooperation between disciplines are indispensable for the effective utilization and innovative development of NAHs. Take the booming CRISPR‐based technology, for example, they have developed to an efficient tool of gene editing from basic researches to clinical applications. Certainly, this superior technology has penetrated in other fields, such as the development of smart DNA hydrogels. In the published work of English et al.,^[^ ^60^ ^]^ they designed a smart DNA hydrogel with the responsiveness of CRISPR‐associated nucleases. This novel biomaterial achieved rapid and sensitive responses to Cas enzymes and promoted the modularity and universality of DNA hydrogel. Similar manners of technology integration can be imitated to give full scope to advantages of both sides for NAHs development, and further, achieve mutual penetration of NAHs in multiple fields. In addition, creating hydrogels for utilization in soft robotics and 3D/4D printing has been a particular focus point for research in biomaterial science. Hamada et al.^[^ ^243^ ^]^ reported a dynamic material powered by artificial metabolism using the simultaneous processes of biochemical synthesis and dissipative assembly, providing a solid foundation for the creation of “artificial” biological systems with dynamic, life‐like capabilities.

Overall, through the efforts of scholars and researchers, it is believed that the smart and functional development of NAHs will be promoted during the evolution of DNA nanotechnology to biomaterial science and that their multifarious applications will provide real and tangible benefits to people in the foreseeable future.

## Conflict of Interest

The authors declare no conflict of interest.
